# Transcriptomic signatures differentiate survival from fatal outcomes in humans infected with Ebola virus

**DOI:** 10.1186/s13059-016-1137-3

**Published:** 2017-01-19

**Authors:** Xuan Liu, Emily Speranza, César Muñoz-Fontela, Sam Haldenby, Natasha Y. Rickett, Isabel Garcia-Dorival, Yongxiang Fang, Yper Hall, Elsa-Gayle Zekeng, Anja Lüdtke, Dong Xia, Romy Kerber, Ralf Krumkamp, Sophie Duraffour, Daouda Sissoko, John Kenny, Nichola Rockliffe, E. Diane Williamson, Thomas R. Laws, Magassouba N’Faly, David A. Matthews, Stephan Günther, Andrew R. Cossins, Armand Sprecher, John H. Connor, Miles W. Carroll, Julian A. Hiscox

**Affiliations:** 1 National Institute of Health Research, Health Protection Research Unit In Emerging and Zoonotic Infections, Liverpool, UK; 2 Centre for Genomic Research, Institute of Integrative Biology, University of Liverpool, Liverpool, L69 7ZB UK; 3 Department of Microbiology, School of Medicine, National Emerging and Infectious Diseases Laboratories, Bioinformatics Program, Boston University, Boston, MA 02118 USA; 4 Heinrich Pette Institute – Leibniz Institute for Experimental Virology, 20251 Hamburg, Germany; 5 Bernhard Nocht Institute for Tropical Medicine, D-20359 Hamburg, Germany; 6 German Center for Infection Research (DZIF), partner site Hamburg, Germany; 7 Institute of Infection and Global Health, University of Liverpool, Liverpool, L69 7BE UK; 8 Public Health England, Porton Down, Wiltshire, SP4 0JG UK; 9 Bordeaux Hospital University Center (CHU) -INSERM U1219- Bordeaux University, Bordeaux, France; 10 Defence Science Technology Laboratories (Porton Down), Porton Down, Salisbury, UK; 11 Hôpital National Donka service des Maladies infectieuses et Tropicales, Conakry, Guinea; 12 School of Cellular and Molecular Medicine, University of Bristol, Bristol, BS8 1TD UK; 13 National Institute of Health Research in Emerging and Zoonotic Infections, Porton Down, SP4 0JQ Salisbury, UK; 14 Médecins Sans Frontières (MSF), Brussels, Belgium

## Abstract

**Background:**

In 2014, Western Africa experienced an unanticipated explosion of Ebola virus infections. What distinguishes fatal from non-fatal outcomes remains largely unknown, yet is key to optimising personalised treatment strategies. We used transcriptome data for peripheral blood taken from infected and convalescent recovering patients to identify early stage host factors that are associated with acute illness and those that differentiate patient survival from fatality.

**Results:**

The data demonstrate that individuals who succumbed to the disease show stronger upregulation of interferon signalling and acute phase responses compared to survivors during the acute phase of infection. Particularly notable is the strong upregulation of albumin and fibrinogen genes, which suggest significant liver pathology. Cell subtype prediction using messenger RNA expression patterns indicated that NK-cell populations increase in patients who survive infection. By selecting genes whose expression properties discriminated between fatal cases and survivors, we identify a small panel of responding genes that act as strong predictors of patient outcome, independent of viral load.

**Conclusions:**

Transcriptomic analysis of the host response to pathogen infection using blood samples taken during an outbreak situation can provide multiple levels of information on both disease state and mechanisms of pathogenesis. Host biomarkers were identified that provide high predictive value under conditions where other predictors, such as viral load, are poor prognostic indicators. The data suggested that rapid analysis of the host response to infection in an outbreak situation can provide valuable information to guide an understanding of disease outcome and mechanisms of disease.

**Electronic supplementary material:**

The online version of this article (doi:10.1186/s13059-016-1137-3) contains supplementary material, which is available to authorized users.

## Background

Ebola virus (EBOV) causes a devastating infection known as Ebola virus disease (EVD) that in many patients leads to a fatal outcome. The virus responsible for the 2013-2016 West African outbreak was caused by a new strain of EBOV called Makona. In the Republic of Guinea, which was at the epicentre of the 2013-2016 EBOV outbreak, the fatality rate was around 60% and higher than in Sierra Leone and Liberia. The processes that lead either to survival or a fatal infection are unknown although viral load (the amount of virus) can be a key determinant [1, 2]. This is especially relevant at extremes where patients with very high viral loads have a poor prognosis. Factors influencing outcome in patients with EVD include hospitalisation (i.e. access to palliative care) [3], anti-viral treatment [4] and age [5].

The interplay between EVD and outcome is complex and involves a balance between the host response and viral load. Analysis of four patients from the 2013-2016 EBOV outbreak who underwent intensive treatment and care at Emory University Hospital in the USA indicated these patients had a robust immune response during the acute phase of EBOV infection [6], which challenged previous assumptions about the ability of EBOV to suppress the immune system [6]. Certainly in non-human primate models of fatal EBOV infection, an extreme aberrant immunological status and anti-inflammatory response was shown to contribute to the development of fatal haemorrhagic fever [7] and this has also been observed together with lymphocyte apoptosis in fatal human infections with EBOV [8]. Analysis of samples from patients with EVD treated in Guinea from the 2013-2016 outbreak also revealed an immune component influenced survival [6, 9].

The study of patient samples taken from previous outbreaks suggests that host responses may delineate survival and fatal outcomes and potential biomarkers indicative of these outcomes can be identified. In the 2000–2001 Sudan-associated EVD outbreak in Uganda, the case fatality rate for paediatric patients was lower than for adults [10]. Data indicated that paediatric patients who survived had differential abundance of certain serum proteins from paediatric patients who died and that, in contrast, adults had similar levels of these same molecules [11]. Whereas fatality rates in the 2013-2016 West African outbreak suggested that children aged less than five years had a poorer prognosis [5].

Apart from providing data on the underlying causes of EVD, being able to predict the outcome of infection based on analyte concentrations would provide guidance as to potential treatment and would also uncover new therapeutic strategies [11, 12]. Several such immunological/biochemical biomarkers have been identified in a previous outbreak of EBOV-Sudan [11, 12]. However, currently, viral load measurements by real-time quantitative polymerase chain reaction (qRT-PCR) (given as a Ct value) are considered the gold standard for predicting EVD outcome. Viral load measured as Ct can give some indication for outcome [1, 13], and has been particularly useful in the triage of patients for experimental anti-viral trials for EBOV [14]. Viral load for predicting outcome works well, particularly at extremes of Ct, where for example a Ct of 12 would be suggestive of a fatal infection and a Ct value of 30 would be indicative of survival. However, it works less well where Ct values are not able to distinguish outcome, mainly between Ct 20 and Ct 22. Here the outcome is approximately equal between survival and a fatal infection.

The 2013-2016 EVD outbreak was unprecedented in scale and has revealed previously unappreciated aspects of EBOV biology, such as persistence in semen [15]. The strain responsible for this outbreak, EBOV Makona, also appears to have different growth kinetics when compared to previous strains, having a delayed onset to disease progression in a non-human primate model of EVD [16] and reduced lethality in an immune-deficient mouse model [17]. Given the potential new strain variation of EBOV responsible for this outbreak and EVD is a disease of the host response, we wanted to investigate this using a genomics approach to identify transcriptome changes in peripheral blood from acute patients who either went on to survive or die from EVD. This would allow us to investigate whether those patients that succumbed to EBOV showed a hyperactive response to infection and also potentially to identify host-based response markers that can help predict survival in situations where viral load gives little predictive value.

Therefore, to characterise the infection of EBOV Makona in the human population, we first used deep sequencing to define the transcriptomic profile of blood taken from acute patients who either went on to survive or die from EVD. These samples were obtained from patients located in Guinea. During the 2013-2016 outbreak, Guinea recorded the highest death rate for EBOV. Thus, EVD patients in Guinea were optimal for correlating changes in the host response to outcome—either a fatal or non-fatal infection. Their responses to EVD would not have been influenced by intensive palliative and/or experimental care that was utilised in high-income countries to treat repatriated healthcare workers, where 81.5% of patients who received supportive care survived [3]. In contrast, the survival rate in Guinea was approximately 40% throughout the outbreak. These transcriptional signatures from acutely ill patients were compared to profiles obtained from former patients who had recovered from EVD and were EBOV-negative by qRT-PCR and also to data obtained from healthy volunteers mined from historical datasets.

Using transcriptome data from acutely infected patients and from the control patients, profiles were identified that highlighted differences in the host response to EBOV at the time of acute infection. These data were obtained for patients who were either going to survive or die from EVD. The profiles of acutely infected individuals differed from the survivor EBOV-negative group and the healthy controls. The profiles provide significant insight into the global circulating immune response to EBOV in acutely infected patients on a scale that has previously not been possible. Significantly, differences in immune cell populations predicted through analysis of gene expression patterns were validated on an independent group of EVD patients using flow cytometry to directly measure the same cell types in patient blood. Machine learning was used to identify a panel of genes whose abundance could be used to predict the outcome of infection at the acute phase. This panel was validated on a separate independent group of patients with either a fatal or non-fatal outcome and whose viral loads were similar and was found to accurately predict outcome. This would be particularly useful in a clinical setting in cases where the viral load provides little to no predictive power.

## Results

### Analysing patient groups with EVD

**Fig. 1:**
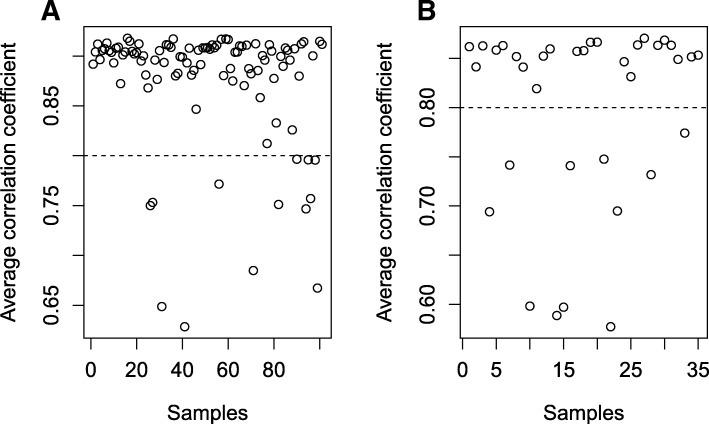
The sample selection criteria based on correlation value to within group expression. A mean correlation within the acute fatal (**a**) and acute survivors (**b**) was used to determine samples with evidence of unreliable sequencing. A cut-off value of 0.8 was used (*dashed line*) and samples that fell below this within-group mean correlation were removed from analysis. This led to selection of 88 acute fatal samples and 24 acute survivors

To identify changes in the host response following exposure to EBOV, the transcriptome of blood samples from infected patients was analysed. These samples were collected by the European Mobile Laboratory in Guinea during 2014 and 2015. The samples were taken with the foremost aim of diagnosing the presence of EBOV using qRT-PCR, which was then used in patient management in the Ebola Treatment Centre (ETC). For this purpose, RNA was extracted in the setting of the European Mobile Laboratory in Guinea. Discarded samples were then held in an archive and used in this study under the auspices of the EVIDENT project– Ebola Virus Disease correlates of protection, determinants of outcome and clinical management. These discarded samples were then analysed by RNA-sequencing (RNA-seq) to identify and quantify messenger RNA (mRNA) expression. Following sequencing of 138 individual samples from individual patients, sample selection criteria were used to identify and remove datasets from samples that showed evidence of having degraded mRNAs (expected from field sample collection) (Table 1). These criteria included the removal of samples that showed poor mapping and low correlation values (Fig. 1 and ‘Methods’). Here, samples were selected that had a similar within-sample transcriptional profile (with an average correlation co-efficient above 0.8), thus reducing extreme variation within one patient group due to mRNA quality issues. From an initial set of 138 individual sequenced samples from separate patients, application of these selection criteria led us to discard 26 and analyse the data from 112 unique patients: acute-survivor (*n* = 24) and acute-fatal (*n* = 88). The outcome for these patients was unknown at the time of sample collection and subsequently recorded as they either succumbed to a fatal infection or survived EVD. These were from a mixture of *Plasmodium* (the causative agent of malaria) negative and positive patients. Importantly, there was no significant difference in the time between symptom onset (as reported by the patient) and taking of the sample during acute illness. The mean time to the onset of symptoms for the acute survivors was 6.4 days and for acute fatalities was 5.9 days with a range of 2–19 and 2–22 days, respectively.

**Table 1:** Details of discarded diagnostic samples from patients used in the study. The numbers in parentheses indicate the number of patients in each group

Sample set	Quality	Survival	Analysis
EBOV-positive samples for RNA-seq (*n* = 138)	Poor quality: removed from analysis (*n* = 26)	N/A	N/A
	Good-quality samples (*n* = 112)	Survived (*n* = 24)	Transcriptomic analysis for differential gene expression and classifier generation
		Fatal (*n*= 88)	
EBOV-positive and malaria-negative (*n* = 37)	N/A	Fatal (*n* = 20)	Flow analysis
		Survived (*n* = 17)	
Febrile and EBOV-negative (*n* = 10)	N/A	N/A	
Malaria-positive and EBOV-negative (*n* = 5)	N/A	N/A	
EBOV-positive sample with similar Ct values (*n* = 20)	Good quality (*n* = 20)	Survived (*n* = 10)	Validation dataset for differential gene expression and classifiers
		Fatal (*n* = 10)	
Convalescent EBOV-negative (*n* = 16)	Good quality (*n* = 16)	Survived (*n* = 16)	Control for EBOV-positive samples
Healthy controls (*n* = 6)	Good quality (*n* = 6)	Never infected (*n* = 6)	Comparator for convalescent samples

To provide a baseline for gene expression, a blood sample was taken from a separate group of 16 survivors that were EBOV-negative by PCR and convalescent for the disease and recovered from infection (recovered control group) (Table 1). This control group represented a critical comparison population. During the 2013-2016 outbreak, amid the breakdown of the in-country healthcare system and the stigma of being seen associated with ETCs, acquiring samples from non-infected patients was not possible. The EBOV survivors represented a known EBOV-negative population that were also tested and confirmed to be *Plasmodium*-negative with no other overt signs of a febrile illness (which could have impacted the results in a control group). We also made use of historical datasets obtained from the RNA-seq analysis of peripheral blood taken from healthy volunteers based in British Columbia (Canada) (*n* = 6) and thus would not have been exposed to EBOV or a range of other pathogens present in West Africa [18] (GEO Number GSE53655) (Table 1).

### Analysis of gene responses of patients that differentiate fate after EBOV infection

**Fig. 2:**
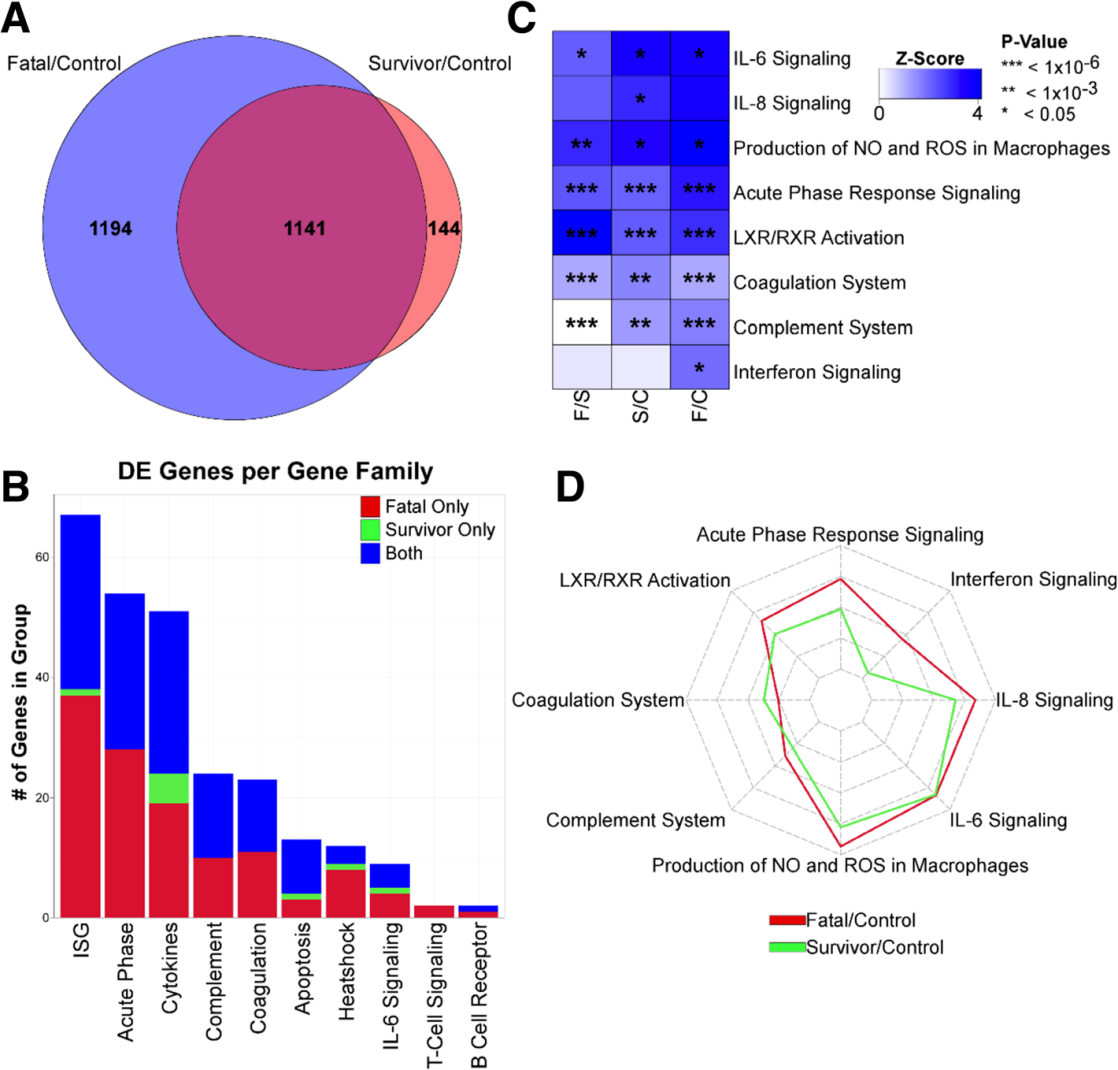
Host transcriptional responses in acute EBOV infection. **a** *Venn diagram* representing genes that are differentially expressed from control to fatal (*blue*) and control to survivor (*red*). Shared genes are shown in *magenta*. **b** *Histogram* illustrating genes increased in abundance in different functional groups. Genes significantly increased in abundance in fatal only (*red*), survival only (*green*) or in both (*blue*) are shown. **c** *Heatmap* of pathway upregulation intensity calculated by Ingenuity Pathway Analysis (IPA) for acute-fatal to convalescent control (F/C), acute-survivor to convalescent control (S/C) and acute-fatal to acute-survivor (F/S). *Stars* within *boxes* represent calculated *p* value significance of increased abundance (* < 0.05, ** < 10^–3^, *** < 10^–6^). **d** *Spider plot* of the z scores from the IPA. Increased abundance in fatal to control is in *red*, survival to control in *green*

We compared the transcriptomes from these different groups to identify mRNAs in survivors and in fatal cases that showed greater than twofold changes (false discovery rate (FDR) 5%) in abundance compared to the recovered control group. In the acute-survivor group, almost 1300 genes were increased in transcript abundance compared to the survivor control group. The corresponding value for the acute-fatal group was 2200 of which half were redundant with those in the acute-survivor group and approximately 1200 were unique to the fatal cases (Fig. 2a and Additional file 1). Additional file 1 shows any gene transcript with an absolute log2 fold change greater than 2 with a FDR of 5%. Most of the gene transcripts were common to both the acute-fatal versus acute-survivor group. Additionally, some gene transcripts that had abundance differences were restricted to the acute-survivor group. Thus, in the blood from individuals with acute EBOV infection, mRNAs of various pro-inflammatory factors such as CXCL10, CCL2/MCP-1, CCL8/MCP2 and CXCL11 showed increased abundance when acute-fatal cases were compared to acute-survivors (Additional file 2). Similar changes in mRNA abundance were observed in a publicly available dataset from non-human primates (NHPs) that survived the challenge with EBOV (Additional file 2).

### Analysis of gene pathways differentiating fate after EBOV infection

Gene set enrichment analysis (GSEA) showed that in acute patients both surviving and having a fatal infection were associated with a significant enrichment of genes from within the same signalling pathways (Fig. 2b). The most significantly represented included gene sets associated with interferon signalling, complement, coagulation, hormone receptor and acute phase signalling. Many interferon-stimulated genes (ISGs) were strongly increased in abundance in all acute infection cases compared to the recovered control group. There was greater increased in abundance of these gene transcripts in acute-fatal compared to acute-survivors.

In addition to GSEA, we also analysed the patterns of differentially expressed genes using Ingenuity Pathway Analysis (IPA) (Qiagen Bioinformatics) to identify signalling pathways associated with infection. Pathways showing significant upregulation in acute infection are shown in Fig. 2c. This analysis identified many of the same pathways identified in GSEA such as complement, acute phase signalling and coagulation factors, but also identified signalling pathways not identified by GSEA, such as IL-6 and IL-8, indicating strong activation of these cytokines in EVD. IL-6 signalling has previously been shown to be associated with fatal EBOV infections in some studies [8, 19] but not others [11]. Our data support the conclusion that IL-6 signalling was upregulated in both the acute-survivor and acute-fatal cases. Spider plot analysis (Fig. 2d) showed that in almost all gene expression categories, acute patients that succumbed to infection showed a more robust immune response.

**Fig. 3:**
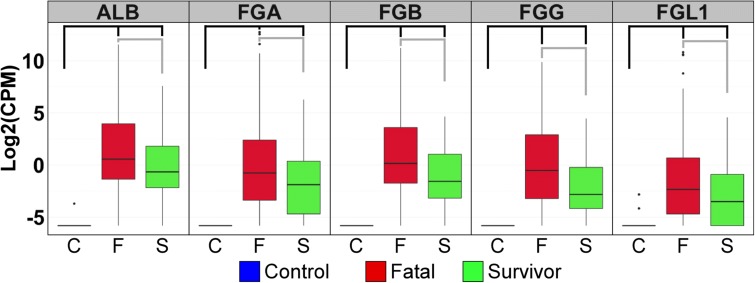
Coagulation associated mRNAs accumulate in the blood of EBOV-infected patients. *Box and whisker plots* illustrate the expression of the top acute phase genes that are differentially expressed between controls (*blue*), fatal (*red*), and survivors (*green*) based on log2 fold change. From *left* to *right*, the *graphs* illustrate mRNA expression for albumin (ALB), fibrinogen alpha (FGA), fibrinogen beta (FGB), fibrinogen gamma (FGG) and fibrinogen gamma-like 1 (FGL1). *Black brackets* indicate significance between control and survivor or fatal levels and *grey brackets* indicate significance level between survivors and those who will succumb to disease. All *brackets* represent a log2(fold change) > 2 and FDR < 5 %

There were also distinctive differences in gene expression patterns when the transcriptomes from acute-fatal cases were compared to those from acute-survivors. A total of 246 transcripts were identified as being differentially abundant (log2FC > 1, FDA 5%), of which 220 transcripts or more increased (higher in fatal cases) and 26 decreased (Additional file 3). This differential abundance was most strongly observed in genes associated with coagulation and acute phase signalling. Figure 3 shows the five most strongly differentially expressed genes, of which four were associated with the clotting cascade genes, including fibrinogen (FG) alpha chain (FGA, +54-fold), beta chain (FGB, +28-fold) and gamma chain (FGG, +19-fold). The increased abundance of these mRNAs is again consistent with deposited transcriptomic data from EBOV-infected NHP studies [20] (Additional file 4).

**Fig. 4:**
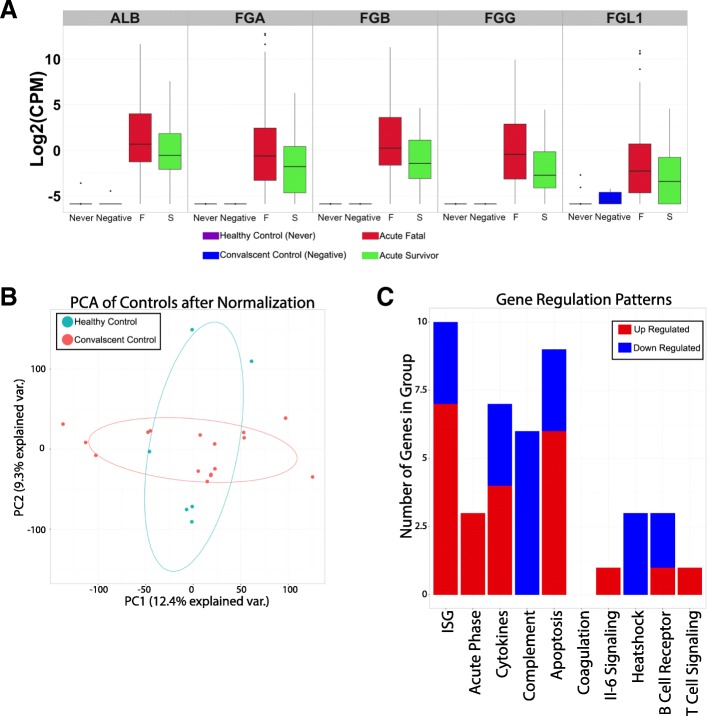
Comparison of the convalescent survivors to healthy controls. The healthy controls comprised a group of healthy volunteers in British Colombia and were found in GEO under GSE53655. **a** The top fold change differentially expressed genes when comparing convalescence survivors (*blue*) to acute infections and when comparing acute survivor (*green*) to acute fatal (*red*). Healthy controls are shown (*purple*). The healthy controls show no significant expression of these genes similar to the convalescent survivors. **b** The comparison of the two control groups as a *PCA plot* of their overall expression values after normalisation using edgeR. The healthy controls are indicated in *blue* and the convalescent survivors in *red*. **c** The results of the gene set enrichment analysis comparing the convalescent survivors to the healthy controls. Genes significantly upregulated are in *red* and downregulated in *blue*. Through a Fisher’s exact test, no group of genes had a significant enrichment when comparing the two groups (*p* cutoff of 0.05)

The transcriptome profile of peripheral blood from the recovered control group may have been influenced by any remaining EBOV antigens and/or other infections (although the patients were *Plasmodium*-negative at the time the peripheral blood sample was taken to confirm convalescence). This may therefore have skewed any transcriptome comparison to the acute patients. To test this hypothesis, we made use of a historical dataset deposited on GEO (GSE53655) that was an analysis of peripheral blood from healthy volunteers [18] (*n* = 6) (Table 1) and compared this to the transcriptome profile from the recovered control group. Given the location of the study for the health volunteers was the University of British Columbia in Canada [18], we assumed that these individuals had not been exposed to EBOV, *Plasmodium* or other pathogens prevalent in Guinea. Differential gene expression (DGE) and GSEA indicated no significant difference in the transcriptome of peripheral blood between the healthy volunteers and the recovered control group (Fig. 4).

### Changing immune cell types in the acute phase are related to patient outcome

**Fig. 5:**
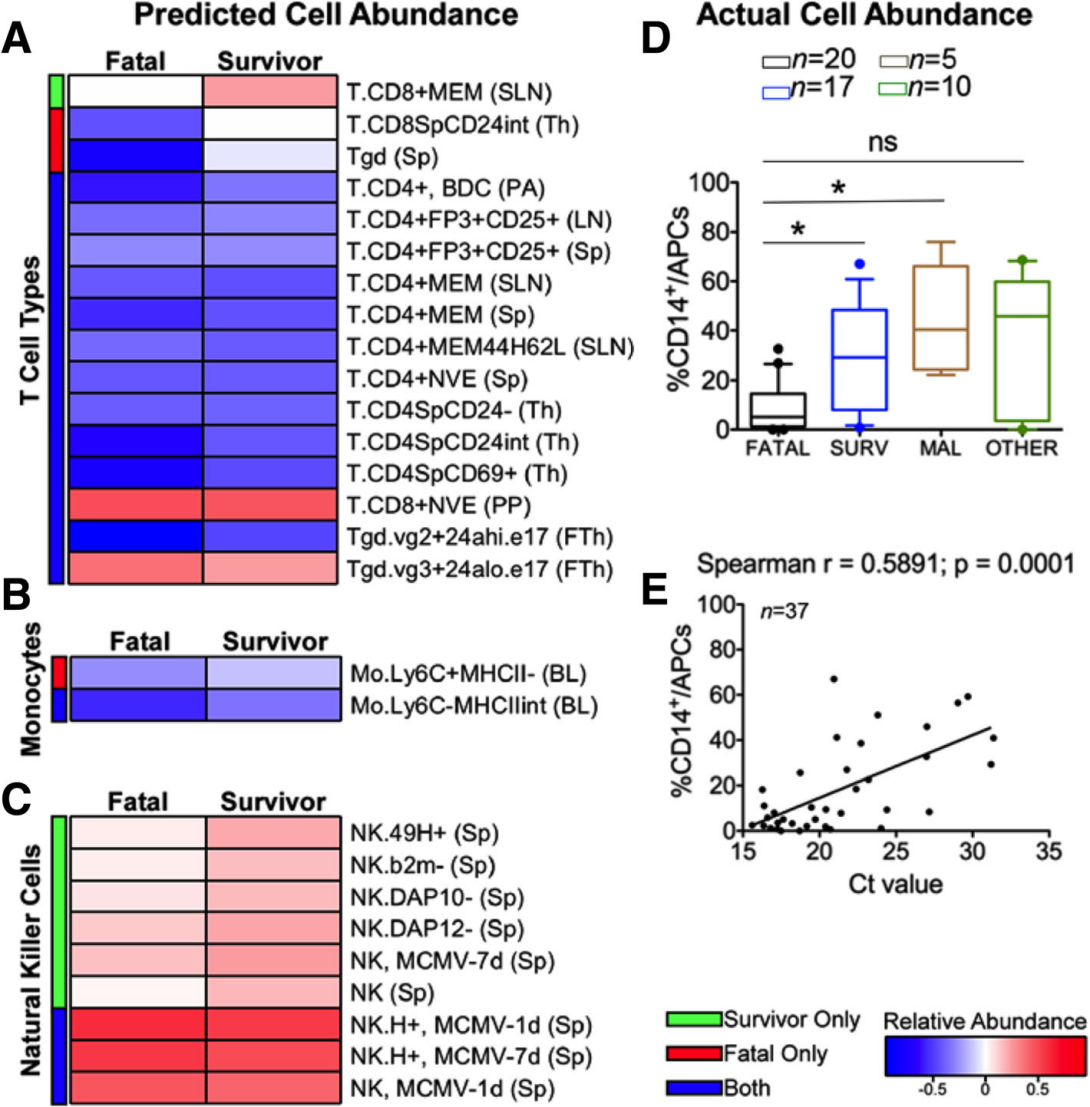
Differentially abundant cell types present in human blood samples. **a** Relative abundance of specific T cell types with acute-fatal on the *left* of the *heatmap* and acute-survivor on the *right* as predicted by DCQ compared to convalescent survivors. Within the heatmap, *darker blue* represents a decrease in the abundance of a given cell type and *darker red* represents an increase in the abundance of a given cell type. The *colour bar* on the *left* is showing if the given cell type is significantly differentially abundant (greater than or less than 0 with a *p* value < 0.05) in acute-survivors only (*green*), acute-fatal only (*red*) or both (*blue*). **b** Similar *heatmap* for dendritic cell types and (**c**) is for natural killer cell types. Loss of circulating monocytes characterises fatal EVD. **d** *Box and whisker plots* depicting frequencies of peripheral blood monocytes in fatal EVD patients (*black*), EVD survivors (*blue*), malaria patients (*brown*) and other febrile patients (*green*) as shown by FACS. *Horizontal bars* represent median values and the edge of the *boxes* represent 10–90 percentiles. Statistical analysis was performed via non-parametric Kruskal–Wallis test followed by Dunn’s post-test. *ns* non-significant, *p ≤ 0.05. **e** Correlation analysis indicating positive correlation between frequency of CD14+ monocytes and the Ct values. Non-parametric Spearman’s rank correlation analysis was performed. The *line* indicates a linear regression

Changes in mRNA abundance in the blood sample between the acute-survivor and acute-fatal patient groups may have been due to changing gene regulation in the cell types present or changing proportions of specific immune cell types. We used digital cell quantification (DCQ) [21] to identify which cell types may have been differentially abundant in acute-survivors and acute-fatal relative to the recovered control group. In both the acute-survivor and acute-fatal groups, DCQ predicted a decrease in CD4 T cells [22] and a significant increase in CD8 memory T cell signature in acute-survivors that is consistent with flow cytometry analysis from patient samples [9] (Fig. 5a and Additional file 5). This analysis also demonstrated a potential decrease in the population of circulating monocytes during acute infection with EBOV (Fig. 5b) with a stronger potential decrease in the acute-fatal compared to the acute-survivor group (Additional file 5).

To investigate the DCQ-based predictions, flow cytometry analysis was used to compare blood samples taken from an independent and geographically separate group of patients (in Guinea) classified as acute-fatal (*n* = 20), acute-survivor (*n* = 17), EBOV-negative but with *Plasmodium* (*n* = 5) and other febrile illness (but EBOV-negative) (*n* = 10) (Table 1). The flow cytometry analysis indicated that in the patients with EVD, who went on to have a fatal outcome, they displayed low frequencies of circulating CD14+ classic monocytes in peripheral blood (Fig. 5d and Additional file 6) compared not only with surviving patients, but also with patients with other acute infectious diseases including malaria (Fig. 4d), consistent with the predictions from the DCQ analysis. In addition, the frequency of blood monocytes in patients positively correlated with viral load, which indicated a statistical association between the loss of circulating monocytes and high viremia (Fig. 5e). Natural killer (NK) cells were predicted to accumulate in acute-survivors when compared to the acute-fatal group (Fig. 5c). Indeed, we predicted that NK cell types were more abundant in acute-survivors than in the acute-fatal group (Fig. 5c), which was consistent with an increase in mRNA abundance in the acute-survivor group of the NK markers interferon gamma (+4 fold over convalescent controls) and perforin (+3 fold over convalescent controls).

### Predictive models based on differentiated gene responses between patient groups

Differential gene expression analysis might provide suitable biomarkers for predicting the eventual outcome of infection in individual patients and thereby better direct their treatment. We assessed if a subset of differential expression (DE) genes could be correlated with outcome using three independent machine learning-based methods. The first method used support vector machine (SVM) analysis. Predictive models based on the top ten genes ranked by *p* value (79% accuracy) performed better than models based on the level of EBOV RNA in a qRT-PCR [14]. However, genes identified through this approach were not linked to known components of the immune response and both models showed wide dispersions for each gene, which contributed to overlaps between the acute-survival and acute-fatal groups (Additional file 7). Including samples from the recovered control group in the analysis improved predictive performance slightly and included some of the invoked genes that showed DE responses to EBOV exposure.

**Fig. 6:**
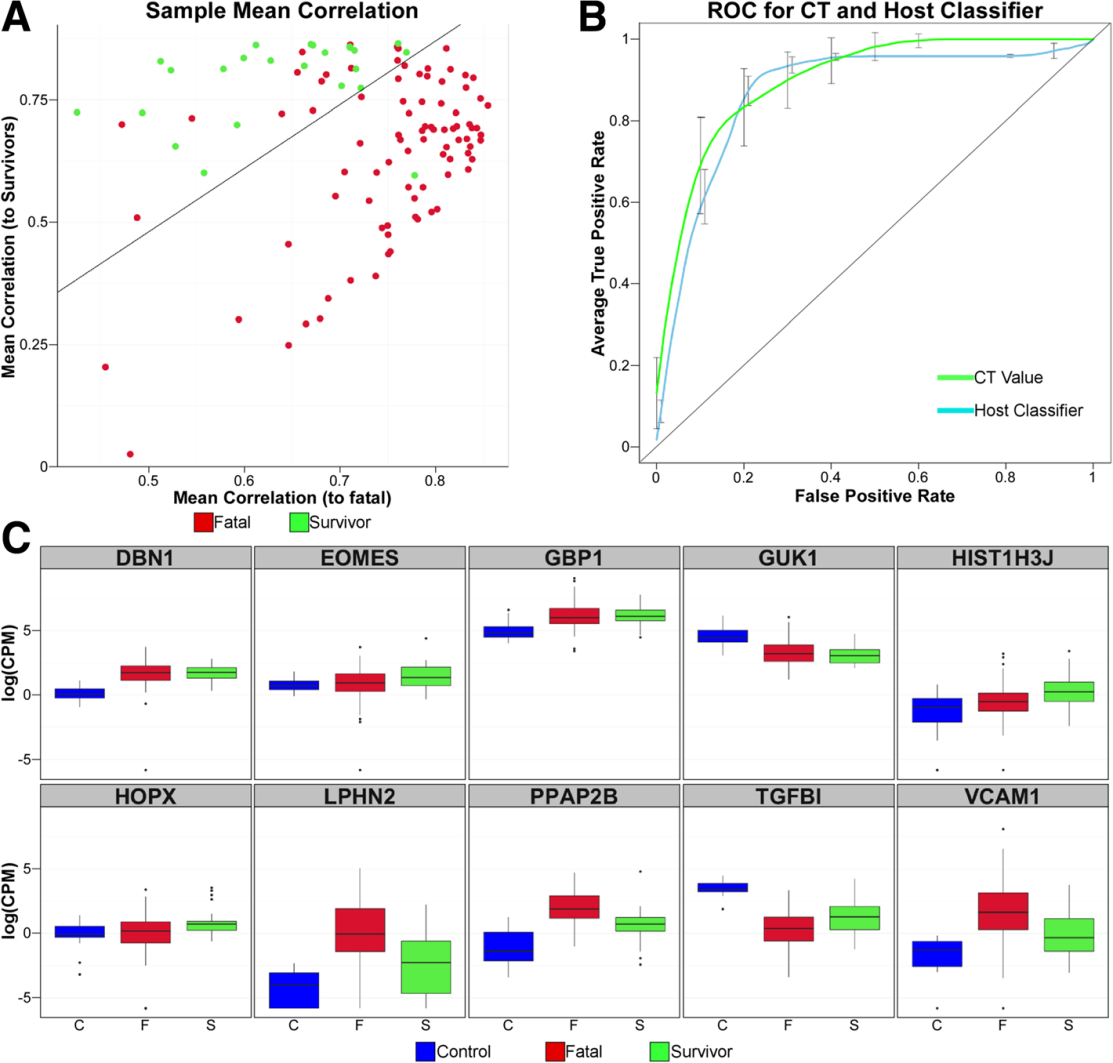
Identification and testing of a small set of host mRNAs whose expression predicts survival during acute EBOV infection. **a** Mean correlation *plots* where each sample is represented. Survivors in *green*, fatal in *red. Line* indicates prediction inflection point. Individuals above the line would be predicted to be survivors using these mRNAs, while individuals below would be predicted to succumb to the disease. **b** *ROC* comparing prediction of survival using EBOV PCR Ct value (*green*) and host mRNA expression classifier (*blue*). The *error bars* represent SD of the average true positive rate. **c** *Box and whisker* plots illustrating expression changes in log2(CPM) for the ten genes in the Host Classifier in control (*blue*), fatal (*red*) and survivors (*green*)

To investigate the predictive power of a host-response-based gene set, a substitution method on groups of ten randomly selected genes was performed to define a better SVM classifier (Figure 6c and Additional file 8). The resulting group of ten genes improved the accuracy of outcome predictions from 79% to 85% (97 of 112 case outcomes predicted correctly). Figure 6a illustrates the resulting separation of acute-fatal cases from instances of acute-survival, while Fig. 6b shows a receiver operating characteristic (ROC) curve compared to Ct. The optimistic bias for the area under the curve (AUC) prediction for this model for the training and testing data was 0.11.

Two other methods were also used to identify gene sets that correlated with outcome. The second method used random forest (RF) and this improved accuracy to 89% (Additional file 8 and 9), again with ten genes as classifiers of outcome. The third method was an entirely different approach and used an intensive optimisation protocol to establish a panel of gene pairs (Additional file 8) for which the consensus expression profile across the panel for acute-survivors were maximally differentiated from the acute-fatal group (‘Methods’, Additional files 10 and 11). We termed this the Paired Gene Profiling method. Classification of a query patient was achieved by correlation of his/her expression profile across the panel to one or other of the alternative classifier profiles. This technique achieved 92% accuracy, using a select group of ten genes that were included within the panel of classifier genes for the improved SVM model (Additional file 8). Thus, the ability to predict outcome was independent of the method used to achieve it.

To test whether these predictions of patient fate were not overly optimistic, we adopted a ‘bootstrapping’ technique. The random resampling of subsets of the overall patient data provides a large number (i.e. 1000) of independent estimates of the predicted status of each patient. The consistency of the outcomes can then be explored from the resulting histogram (Additional file 9), which if normally distributed, as indicated by the linearity of the corresponding QQplot (Additional file 9), is described by a mean ± 95% confidence interval (CI). We show for each of the different predictive methods employed that the distributions were well described by a normal distribution, and that the 95% CIs for predictive accuracy for the RF test were 0.758–0.876, while those for the paired gene profiling (PGP) test were 0.793–0.953. These all lay within ± 8% of the corresponding mean values. Given the absence of data for independent validation, this outcome offers good support to a highly consistent predictive accuracy across the different models.

### Effects of viral load on predictive outcome and testing on a separate group of patients with EVD

**Fig. 7:**
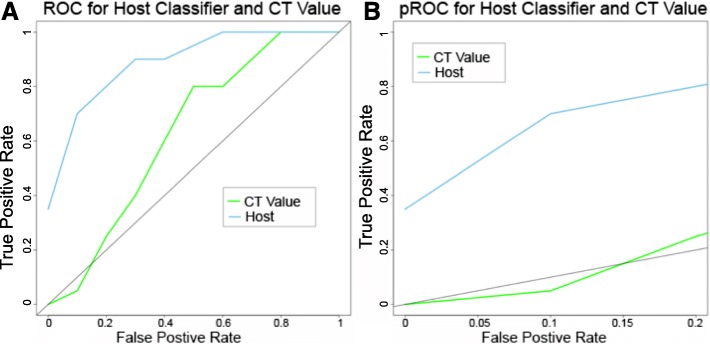
Validation of classifier in an independent dataset. The ability of the host-based classifier to predict outcome was tested in an independent dataset where the Ct values all lay between 20 and 22. **a** *ROC* showing the host-based classifier (*blue*) in the independent dataset compared to the Ct value (*green*). The *line* represents the line y = x which shows where the ROC falls if the predictions are equal to random selection. **b** *pROC* showing the comparison for the host classifier (*blue*) and Ct value (*green*) with a false positive rate up to 0.2. In the validation dataset, the host classifier was able to predict better than the Ct value

The DGE analysis and gene classifiers were generated from patients that had a diverse range of viral load. We wanted to examine whether both were independent of viral load and instead just reflected the host response to EBOV infection. To investigate this, we determined the transcriptome of blood samples taken from an independent group of patients from that used in the initial DGE analysis and for gene classifiers. We focused specifically on samples from new patients whose viral loads were not significantly different (Ct = 20–22) between a fatal (*n* = 10) or non-fatal outcome (*n* = 10) (Additional file 12). This is a crucial range for viral load as between these Ct values the outcome of EVD cannot be predicted as the case fatality rate in this group of patients was approximately 50% from the outbreak in Guinea. From a disease biological perspective, this allowed us to investigate whether the observed differentially expressed genes between a fatal and survivor outcome were potentially related to viral load. For example, a lower viral load promoted survival or whether DGE was potentially host-moderated. All of these selected patients were antigenically negative for *Plasmodium* and thus the outcome and the host response were not complicated by malaria. DGE analysis between these two groups of patients identified the enrichment of very similar transcript abundance to that described in the original DGE analysis on the acute-fatal and acute-survivor patients groups (Additional file 13), suggesting that the host response (at least at the transcript level in the blood) was mainly independent of viral load. There was also good correlation between the top five differentially abundant gene transcripts expressed in the *n* = 20 study and the *n* = 112 patient study (Additional file 14). When the ten gene SVM classifier was compared to the Ct value in this group, it greatly outperformed the Ct value in predicting survival as seen in the ROC and partial (pROC) in Fig. 7. The ten gene RF classifier was also able to predict survival in this group of patients where the Ct value failed. This shows that the ability of the biomarker to predict outcome was independent of Ct value and is useful predicting outcome in patients where the Ct value is uninformative.

## Discussion

Understanding the pathogenesis of rare outbreak diseases such as EBOV is both difficult and important. To date, the overwhelming majority of cases of this disease have occurred in situations where high-quality healthcare and monitoring are challenging. This has made it difficult to understand in detail many of the basic aspects of disease development and pathogenesis; information on the disease has been gathered through animal models [16, 17].

Here we show that transcriptomic sequencing of blood samples taken from humans during the 2013-2016 West African outbreak and leftover from diagnostic sequencing can be an important means of acquiring multiple levels of information about the host response to virus infection, ranging from understanding how immune cell populations in the blood change over time during infection to helping define potential host biomarkers of infection. This strongly argues that integrating transcriptomic analysis of host responses during outbreaks can provide important insights into disease pathogenesis that affect clinical management. The strong ISG response observed in the large cohort of EVD patients is notable. IFN-like responses have been reported to be associated with moderate disease in a diverse set of US EBOV patients [23] and have been suggested to be protective for EBOV infection [24]. The existence of a strong innate immune response in acute cases in Guinean patients, given their differing clinical outcome and treatment, suggests that this response may not be offering significant protection to promote survival.

A particularly interesting finding from this study was the effectiveness of using a recovered control group as a comparison population to investigate differential gene expression. Ideally, a control group from Guinea that was never infected with EBOV would have been optimal; however, at the time (and still persisting), stigma associated with EBOV and ETCs rendered such a group challenging to identify and ethically problematic to take blood samples. Using recovered control group transcriptomes had the potential to identify some responses that began during acute Ebola infection and had not been resolved when samples were taken from the control group. However, comparison of the peripheral blood transcriptomes from this group to historical datasets from a completely unrelated healthy control group separated both geographically and temporally from the West African outbreak [18] indicated no significant differences in the transcriptome (Fig. 4). This suggested that at least when the peripheral blood samples were taken the control group had recovered from infection. We find that immune-cell populations predicted by our comparison analysis (Fig. 5) and biomarkers identified through comparison were both verified in independent datasets (e.g. Fig. 6 and Additional file 13). It is unlikely that the Ebola outbreak will be the only one in which the ‘ideal’ control population is difficult to sample and our results suggest that convalescent patients may serve as an important alternate control. By necessity, datasets from samples that had poor-quality reads were excluded from the analysis presented in this work. This may have biased the identification and measurement of differentially expressed genes as well as the downstream analysis of gene classifiers that correlated outcome. However, the underlying biology of EVD identified in this study through the transcriptomic approach correlated with both data from non-human primate studies and clinical information from patients.

The increased accumulation of mRNAs for genes involved in the clotting cascade found during acute infection with EBOV is consistent with earlier findings that fibrin deposition was closely associated with EBOV infection [25, 26]. The increased abundance of multiple FG gene isoforms as well as the increased abundance of albumin mRNA is initially perplexing, as these genes are considered liver-specific mRNAs and not mRNAs that are found in blood. We favour the hypothesis that the accumulation of these genes is an indication of significant liver damage leading to leakage of hepatic mRNAs into the blood [27]. It is important to note that during this outbreak, overt haemorrhaging was rarely observed [28], but strong increases in the abundance of these genes was seen in both the acute-survivor and acute-fatal patient groups, suggesting that significant liver damage was present. As a measure of aberrant liver function, in patients with EVD, aspartate transaminase (AST) values were found to be higher than alanine transaminase (ALT) [29]. This finding is consistent with observations of liver damage in repatriated patients from Liberia treated in the United States [30].

An important finding of this study is the similarity of the transcriptomic data to protein expression data analysing cytokine expression observed in this [9] and earlier outbreaks [8]. These data emphasise the similarity of cytokine abundance information collected through this approach to that seen in previous EBOV outbreaks and also to NHP models of EVD in humans (Additional file 2). The robust IFN response seen in human infection was somewhat surprising based on earlier reports. However, this was consistent with data from NHP models of lethal infection with this virus [31] that also show very strong increases in IFN-responsive genes in circulating immune cells and suggests that more robust IFN signalling may decrease an individual’s ability to survive EBOV infection. Certainly, our study on patients treated in a low-income setting and results from patients treated in a high-income setting [6] challenges the assumption that humans do not mount a robust immune response.

The changes in the immune response identified in this study in the acute phase of EVD between acute-survivors and acute-fatal patients were potentially caused by the differential activation of gene transcription and also potential infiltration/exfiltration of different cell types in the blood. Our prediction that monocyte cell populations were higher in the acute-survivors than the acute-fatal patients was validated on an independent group of patients using a cell-based approach (Fig. 5d)—completely different to that of RNA-seq (Fig. 5a and Additional file 6). We also predicted that NK cell populations were higher in acute-survivors compared to acute-fatal patients during the acute phase. NK cells have been previously suggested to be important innate immune cells in fighting EBOV infection [32], so the increased abundance of these cells perhaps providing the crucial survival advantage to the acute-survivors group.

Independent machine learning approaches identified different panels of genes whose abundance could accurately predict outcome over a range of Ct values. Using host gene profiles to predict outcome also worked for those Ct values (between 20 and 22) where the outcome was not clear in the data from the European Mobile Laboratory, i.e. where the case fatality rate was approximately 50%. Other studies have also shown that Ct values can be used to predict outcome. For example, a study of EVD patients in Sierra Leone showed that patients with a Ct ≥ 24 had an 87% chance of survival, whereas patients with a Ct value < 24 had a 22% chance of survival [2]. Interestingly, the average patient Ct value in samples processed by the European Mobile Laboratory was 21.4, implying that for the average patient their outcome could not have been predicted based on Ct value alone. We show that the identified gene classifiers were valid over a wide range of viral loads which clearly indicated that the host response was unrelated to the amount of viremia. The classifiers were tested on an independent group of 20 patients. This provided strong preliminary information that the model was not highly over-fitted, though additional samples would increase confidence.

Assessing viral load together with an evaluation of the host response at the time of diagnostic sampling may give an accurate indication of the survival chance for the patient, across a broad range of viral loads. Management of patients in the developed world resulted in far better survival rates than the management of patients in West Africa, due to extensive palliative intervention; therefore, the acute and fatal outcomes in this study may be more reflective of the situation in untreated patients. The triage of large numbers of patients under very resource-poor situations may promote survival rates by focusing efforts on those most in need. Predictive models based on clinical data have also been proposed to determine the outcome for patients with EVD [33].

The ability to triage patients by disease severity and likely outcome can be of practical benefit for patient care. In any outbreak setting, it is inefficient to have the resources for the most intensive care scattered about the Ebola Treatment Unit (ETU). Developing tests that allow the stratification of risk would allow an ETU to centralise intensive care resources for maximum efficiency and efficacy.

Our findings also have implications for the design of clinical trials. Recent studies used Ct value as a proxy for the probability of survival (e.g. the Favipiravir trial), but as we demonstrate here, Ct-based prediction is not perfect. As our results provide improved prediction of outcome, the potential to show an effect of a therapeutic through a clinical trial is improved. During the last outbreak, there was much public disagreement over the ethics of randomising patients with EVD to control groups in clinical trials [34–36]. In our opinion (and that of others), a reasonable compromise position would be to exclude from randomisation those patients with the lowest probability of survival and provide them with the study drug. The results of our work would allow for a more accurate identification of those EVD patients with a low probability of survival rather than simply rely on Ct values at admission.

The mRNAs associated with the correct prediction of outcome include the transcription factor eomesodermin (eomes), an important characteristic of CD8 T cell memory transition [37], and is consistent with our prediction of increased CD8+ memory cells in survivors (Fig. 5). Consistent across the three gene sets identified by SVM, RF and the PGP profile-based classifier were TGFB1, VACM1 and HOPX. TGFBI is an extracellular matrix protein that inhibits cell adhesion and is seen to be downregulated in both fatal patients and survivors. VCAM1 is a gene important for lymphocyte extravasation to sites of infection. This gene has previously been shown to be upregulated in response to EBOV infection [38], consistent with our prediction of increased CD8+ memory cells in survivors (Fig. 5). The decreased abundance of TGFB1 and the increased abundance of VCAM1 is suggestive of an increase in cell adhesion with an increased instance of leukocyte cell adhesion to the endothelial layer and movement out of the blood into tissues.

## Conclusions

These data demonstrate that a blood sample from a patient with EVD is a powerful source of disease information, which is relevant to clinical treatment. Analysing the population of mRNAs present in the blood provided information on the extent of the immune response to infection, the immune cells engaged (and depleted) during infection, and also the accumulation of mRNAs likely derived from damaged internal organs such as the liver. The changes in mRNA abundance also proved to be a reliable means of predicting whether an individual patient would survive or succumb to EVD.

In large-scale outbreaks, blood represents one of the most available and informative of diagnostic samples. Our studies suggest that using collected blood to analyse how a patient is responding to infection will allow better diagnosis and prediction of disease outcome for this highly fatal infection. Though the analysis of a patient’s response to infection has not always been thought of as an important component of a diagnostic approach, this report and others [39–41] show that it has significant value and likely should be adopted into future diagnostic and treatment approaches.

It is important to note that the differences between patient groups are likely to have been due to a mixture of gene regulation and changes in the cell types represented in the blood samples. This complexity offers opportunities to identify cell populations recruited to successfully combat infection. Our analysis supports the hypothesis that in acute patients who survive infection, gene transcripts associated with the presence of NK cells may play a major role in outcome, suggesting that approaches to enhance this population of cells could be an effective EVD treatment strategy.

Integration of this type of analysis in future outbreak responses could help direct therapy and maximise a beneficial outcome of infection, particularly in the development of diagnostic approaches that can accurately stratify patients for treatment based on the likely outcome of infection.

## Methods

### RNA extractions, library preparation and sequencing

Samples from patients were sequenced on a HiSeq2500 and several criteria were applied to the selection of data post sequencing. A total of 38,554 genes were mapped to the 64,253 genes in the annotated human genome database. The RNA was DNase treated using Ambion Turbo DNase. RNA–seq libraries were prepared from the DNAse-treated RNA using the Epicentre ScriptSeq v2 RNA-Seq Library Preparation Kit and following 10–15 cycles of amplification; libraries were purified using AMPure XP beads. Each library was quantified using Qubit and the size distribution assessed using the Agilent 2100 Bioanalyser and the final libraries were pooled in equimolar ratios. The quantity and quality of each pool was assessed by Bioanalyzer and subsequently by qPCR using the Illumina Library Quantification Kit from Kapa on a Roche Light Cycler LC480II according to the manufacturer’s instructions. The template DNA was denatured according to the protocol described in the Illumina User Guide and loaded at 12 pM concentration. To improve sequencing quality control, 1% PhiX was spiked-in. The sequencing was undertaken on the Illumina HiSeq 2500 with version 4 chemistry generating 2 × 125 bp paired-end reads. Base calling and de-multiplexing of indexed reads was performed by CASAVA version 1.8.2 (Illumina) to produce all the sequence data in fastq format. The raw fastq files were trimmed to remove Illumina adapter sequences using Cutadapt version 1.2.1 [42]. The option ‘-O 3’ was set, so the 3′ end of any reads which matched the adapter sequence over at least 3 bp was trimmed off. The reads were further trimmed to remove low-quality bases, using Sickle version 1.200 with a minimum window quality score of 20. After trimming, reads shorter than 10 bp were removed. Trimmed R1/R2 read pairs were mapped to the human reference genome assembly GRCh38 (ftp://ftp.ensembl.org/pub/release-77/fasta/homo_sapiens/dna/Homo_sapiens.GRCh38.dna_sm.primary_assembly.fa.gz) using TopHat2 version 2.1.0 [43, 44], which calls the mapper Bowtie2 version 2.0.10 [44]. Paired-end mapping was carried out using option ‘-g 1’, ‘--library-type fr-secondstrand’, ‘--mate-inner-dist 160’ and ‘--mate-std-dev 60’, which instructs TopHat2 to allow a maximum of one alignment to the reference for a given read, choosing the alignment with the best alignment scores if there is more than one or discarding the read if there is more than one equally good alignment.

The alignments were used for calculating read counts per gene using HTSeq-count (http://www-huber.embl.de/users/anders/HTSeq/doc/count.html). The raw counts generated from HTSeq-count were imported into the R environment to carry out differential expression analysis using edgeR [45] for contrasting acute when tested and survived (acute survivor), acute when tested and died (acute fatal) and acute (either fatal or survived) compared to convalescent controls. Differentially expressed genes with a FDR < 5% and an absolute log_2_ fold change > 1 were finally reported. Details on the sample numbers are provided in the text and in Table 1.

### Informatic analysis

Three criteria were used to identify sample quality: (1) removal of low-quality samples from the dataset such that all samples eventually selected had over ~10% of reads mapping to the human genome; (2) calculation of the correlation coefficient of each remaining sample against any other samples within each groups and the scatter plot of all correlation coefficient values was plotted for the fatal and survivor groups; and (3) a cutoff of a mean correlation coefficient of 0.8 was selected based on the distribution and any samples with a mean correlation coefficient of less than 0.8 were eliminated.

To identify gene expression signatures for predicting outcomes of Ebola patients, a machine learning approach was used (SVM) function in R package ‘e1071’ [46]. These were based on the ten genes either with the highest fold change or most significant identification. The performance of predictions was assessed following a leave-one-out cross-validation approach. The prediction performances were assessed by using R package ‘ROCR’ [47] through ROC curves and Accuracy-FPR (False Positive Rate) plots (see Additional file 7).

To perform the expansion substitution method to identify an optimal classifier, first, the total gene set was reduced to a search space containing only genes that were differentially expressed from convalescent controls to acute-fatal or convalescent controls to acute-survivors. Then, for each iteration, ten genes were selected at random to be the starting profile set. Each gene in the search space was substituted for a profile gene and the correlation within groups (acute-fatal to acute-fatal and acute-survivor to acute-survivor) and between groups (acute-fatal to acute-survivor) was calculated. If the new gene substitution improved the within-group correlation and decreased the between-group correlation, the new profile set was selected. This was run until convergence was reached and a last profile gene set was generated. The whole process was run 50 times. The final profile set included the genes that showed up the most in a last gene set. Finally, the accuracy of the classifier was determined by leave-one-out cross-validation where a sample was classified as acute-survivor or acute-fatal based on the correlation to the acute-fatal group or the acute-survivor group. The bias estimations were performed using the boot function in R for 100 iterations. A cutoff value for the correlation was determined at each cv step and if the sample fell below the cutoff line, it was classified as acute-fatal and above acute-survivor. Training and testing of the model was performed in the original dataset of 112 samples. The trained model was then used in a validation dataset of 20 independent samples.

### Ingenuity Pathway Analysis

The *p* value was calculated by Fisher’s exact test right-tailed, which indicated the probability of association of molecules from the dataset with the canonical pathway by random chance alone. The Z-score was used to mathematically compare the dataset with the canonical pathway patterns, taking into account the activation state of one of more key molecules when the pathway was activated and also the molecules’ causal relationships with each other. This is a Quant-based test that determined if canonical pathways, including functional endpoints, were increased or decreased based on differentially expressed genes or proteins in the dataset.

### Digital cell quantification

To determine relative cell type quantities from count data, we used DCQ through the R-package ComICS [21]. The default parameters were used with 500 repeats and a split ratio of 50% on an input dataset of fold changes compared to controls (acute-fatal to controls and acute-survivors to controls). All 207 cell types were analysed. The resulting data provided a predicted mean relative abundance as well as the standard deviation of the mean. To determine cell types that were significantly different from zero, a standard t-test was performed with a *p* value cutoff of 0.05. Definition of immune subsets is based on ImmGen database information and thus represents previously validated mouse equivalents to human immune cells [48–50].

### Study samples for flow cytometry analysis

RT-PCR was performed on EDTA-blood of patients with suspected EVD using the RealStar Zaire Ebolavirus RT-PCR Kit 1.0 (Altona Diagnostics). EVD-positive patients (*n* = 37) included in the study were diagnosed by the European Mobile Laboratory unit at the ETC Coyah and were medically attended by medical teams deployed by the Cuban government. Patients with *Plasmodium* co-infection were excluded from the study. Additionally, ten febrile EVD-negative controls and five patients diagnosed with malaria were included in the study. Malaria was diagnosed using a rapid test. Leftover samples from diagnostics were shipped within 24 h after collection to our immunology laboratory at Donka Hospital in Conakry and processed immediately. Peripheral blood mononuclear cells (PBMCs) were isolated after red blood cell lysis (BD Biosciences). Immune phenotyping was done by flow cytometry using the following antibodies: CD16-PE (3G8), CD3-PerCP/Cy5.5 (SK7), CD19-PerCP/Cy5.5 (HIB19), CD56-PerCP/Cy5.5 (HCD56), HLA-DR-PE/Cy7 (L243), CD14-APC (HCD14), CD11c-PB (Bu15), CD14-BV510 (M5E2), CD141-PE (M80), CD1c-APC (L161), CD16-APC/Cy7 (3G8). All antibodies were from Biolegend. Live/Dead cell discrimination was done with Zombie NIR staining (Biolegend). After Live/Dead cell staining, PBMCs were treated with FACS block (Human TruStain Fc receptor blocking antibodies from Biolegend) for 20 min followed by extracellular antibody staining. Samples were afterwards inactivated in Cytofix/Cytoperm (BD) buffer in the presence of 4% formaldehyde. Sample acquisition was done in a Guava easyCyte 8 Flow Cytometer from Millipore. Flow cytometry analysis was done with FlowJo software (Treestar). Non-parametric statistics was performed in Graphpad Prism software as described in the legend to Fig. 3.

### Human DCQ

The human cell compendium for DCQ used a microarray dataset of FACS-separated human immune cells (GSE24759). The data were read into R and normalised using the ‘affy’ package and RMA normalisation. Then, the log normalised values for each cell type were averaged for each gene and each gene was then normalised by the median and standard deviation. A list of surface markers was selected based on the FACS separating markers used and other known surface markers for the given cell type. This list of surface markers and the new normalised dataset was read into DCQ along with the fold change data. To determine significance, a standard *t*-test was performed on the mean and standard deviation of the output.

### Non-human primate data

The NHP data were taken from GEO GSE64538. This dataset contained RNA-seq in PBMCs from vaccinated and unvaccinated animals at various time points during viral challenge. Data were read into R and using edgeR and normalised for library size. DE data were calculated using the negative binomial. Fold changes were calculated by first normalising to the pre-infection control for vaccinated and unvaccinated animals and then taking the unvaccinated 7 dpi animals over the vaccinated 7 dpi.

### RF method for phenotypic prediction applied to the Ebola dataset

The list of all genes was split into six strata and the R package ‘randomForest’ was used on expression data for each strata, with patients’ status marked as either Survivor or Fatal. Classification was undertaken separately for the six strata and the data were pooled and genes were ranked to identify the top ten genes which were used as biomarker genes. Finally, the patients’ outcome prediction based on the top ten genes was performed using the ‘randomForest’ method.

### A novel PGP method for phenotypic prediction based on the optimised selection of representative multi-gene expression profiles

See Additional file 11 for a full description of this method.

### Bootstrapping

Bootstrapping for both RF and PGP predictive models was deployed using the R function ‘boot’ with 1000 re-samplings set to generate 1000 estimates of prediction accuracy. Bootstrapping was set in ‘non-parameter’ mode and patient outcome was presented in the function argument ‘strata’.

The bootstrapping results are depicted in Additional file 9, with the left panel providing a histogram of prediction accuracy over the 1000 replications. The right panel shows the QQplot of prediction accuracy against the standard normal distribution, the linearity indicating the extent to which the accuracy distribution is represented by a normal distribution.

## Additional files

Additional file 1: Gene expression profiles of fatal and survivors with EVD compared to a control group. (XLSX 335 kb)
Additional file 2: Fold change of the abundance of selected cytokines showing comparison between fatal or survivor groups between humans (using our datasets) and non-human primate models of infection (using historical published data). (DOCX 184 kb)
Additional file 3: Expression profile differences in acute patients who either went on to have a fatal outcome or survive infection. (XLSX 74 kb)
Additional file 4: Acute phase response genes activated in a non-human primate model of EBOV infection. (DOCX 159 kb)
Additional file 5: DCQ analysis of predicted immune cell type profiles in acute patients who either went on to have a fatal outcome or survive infection. (DOCX 314 kb)
Additional file 6: Flow cytometry gating strategy for discrimination of classic CD14+ peripheral blood monocytes. (DOCX 412 kb)
Additional file 7: Receiver operating characteristic (ROC) curves for p- value host classifier genes versus Ct value. (DOCX 49 kb)
Additional file 8: Genes in the final profile sets for the machine learning approaches. (DOCX 103 kb)
Additional file 9: Outcome of predictive models for the Random Forest method. (PDF 201 kb)
Additional file 10: The paired gene profiling method for outcome prediction. (PDF 386 kb)
Additional file 11: A novel ‘gene paired profiling’ method for improved phenotypic prediction based on the optimised selection of representative multi-gene expression profiles. (DOCX 160 kb)
Additional file 12: Comparison of Ct values between the training and validation datasets. (DOCX 189 kb)
Additional file 13: Gene set enrichment analysis on validation dataset. (DOCX 333 kb)
Additional file 14: Box and whisker plot for the top differentially expressed genes in validation dataset. (DOCX 134 kb)

## References

[1] Carroll MW, Matthews DA, Hiscox JA, Elmore MJ, Pollakis G, Rambaut A (2015). Temporal and spatial analysis of the 2014-2015 Ebola virus outbreak in West Africa. Nature..

[2] Crowe SJ, Maenner MJ, Kuah S, Erickson BR, Coffee M, Knust B (2016). Prognostic indicators for Ebola patient survival. Emerg Infect Dis..

[3] Uyeki TM, Mehta AK, Davey RT, Liddell AM, Wolf T, Vetter P (2016). Clinical management of Ebola virus disease in the United States and Europe. N Engl J Med..

[4] Trad MA, Naughton W, Yeung A, Mazlin L, O'sullivan M, Gilroy N, Fisher DA, Stuart RL. Ebola virus disease: an update on current prevention and management strategies. J Clin Virol. 2017;86:5-13. doi:10.1016/j.jcv.2016.11.005. Epub 2016 Nov 11. 10.1016/j.jcv.2016.11.005 27893999

[5] Shah T, Greig J, van der Plas LM, Achar J, Caleo G, Squire JS (2016). Inpatient signs and symptoms and factors associated with death in children aged 5 years and younger admitted to two Ebola management centres in Sierra Leone, 2014: a retrospective cohort study. Lancet Glob Health..

[6] McElroy AK, Akondy RS, Davis CW, Ellebedy AH, Mehta AK, Kraft CS (2015). Human Ebola virus infection results in substantial immune activation. Proc Natl Acad Sci U S A..

[7] Ebihara H, Rockx B, Marzi A, Feldmann F, Haddock E, Brining D (2011). Host response dynamics following lethal infection of rhesus macaques with Zaire ebolavirus. J Infect Dis..

[8] Wauquier N, Becquart P, Padilla C, Baize S, Leroy EM (2010). Human fatal Zaire Ebola virus infection is associated with an aberrant innate immunity and with massive lymphocyte apoptosis. PLoS Negl Trop Dis..

[9] Ruibal P, Oestereich L, Ludtke A, Becker-Ziaja B, Wozniak DM, Kerber R (2016). Unique human immune signature of Ebola virus disease in Guinea. Nature..

[10] Mupere E, Kaducu OF, Yoti Z (2001). Ebola haemorrhagic fever among hospitalised children and adolescents in northern Uganda: epidemiologic and clinical observations. Afr Health Sci..

[11] McElroy AK, Erickson BR, Flietstra TD, Rollin PE, Nichol ST, Towner JS (2014). Biomarker correlates of survival in pediatric patients with Ebola virus disease. Emerg Infect Dis..

[12] McElroy AK, Erickson BR, Flietstra TD, Rollin PE, Nichol ST, Towner JS (2014). Ebola hemorrhagic Fever: novel biomarker correlates of clinical outcome. J Infect Dis..

[13] Fitzpatrick G, Vogt F, Moi Gbabai OB, Decroo T, Keane M, De Clerck H (2015). The contribution of Ebola viral load at admission and other patient characteristics to mortality in a Medecins Sans Frontieres Ebola Case Management Centre, Kailahun, Sierra Leone, June-October 2014. J Infect Dis..

[14] Sissoko D, Laouenan C, Folkesson E, M’Lebing AB, Beavogui AH, Baize S (2016). Experimental treatment with Favipiravir for Ebola Virus Disease (the JIKI Trial): A historically controlled, single-arm proof-of-concept trial in Guinea. PLoS Med..

[15] Deen GF, Knust B, Broutet N, Sesay FR, Formenty P, Ross C (2015). Ebola RNA persistence in semen of Ebola Virus Disease survivors - preliminary report. N Engl J Med.

[16] Marzi A, Feldmann F, Hanley PW, Scott DP, Gunther S, Feldmann H (2015). Delayed disease progression in cynomolgus macaques infected with Ebola virus Makona strain. Emerg Infect Dis..

[17] Smither SJ, Eastaugh L, Ngugi S, O’Brien L, Phelps A, Steward J (2016). Ebola virus Makona shows reduced lethality in an immune-deficient mouse model. J Infect Dis..

[18] Shin H, Shannon CP, Fishbane N, Ruan J, Zhou M, Balshaw R (2014). Variation in RNA-Seq transcriptome profiles of peripheral whole blood from healthy individuals with and without globin depletion. PLoS One..

[19] Hutchinson KL, Rollin PE (2007). Cytokine and chemokine expression in humans infected with Sudan Ebola virus. J Infect Dis..

[20] Barrenas F, Green RR, Thomas MJ, Law GL, Proll SC, Engelmann F (2015). Next generation sequencing reveals a controlled immune response to Zaire Ebola virus challenge in cynomolgus macaques immunized with VSVΔG/EBOVgp. Clin Vaccine Immunol..

[21] Altboum Z, Steuerman Y, David E, Barnett-Itzhaki Z, Valadarsky L, Keren-Shaul H (2014). Digital cell quantification identifies global immune cell dynamics during influenza infection. Mol Syst Biol..

[22] Reed DS, Hensley LE, Geisbert JB, Jahrling PB, Geisbert TW (2004). Depletion of peripheral blood T lymphocytes and NK cells during the course of ebola hemorrhagic Fever in cynomolgus macaques. Viral Immunol..

[23] McElroy AK, Harmon JR, Flietstra TD, Campbell S, Mehta AK, Kraft CS (2016). Kinetic analysis of biomarkers in a cohort of US patients with Ebola virus disease. Clin Infect Dis..

[24] Smith LM, Hensley LE, Geisbert TW, Johnson J, Stossel A, Honko A (2013). Interferon-beta therapy prolongs survival in rhesus macaque models of Ebola and Marburg hemorrhagic fever. J Infect Dis..

[25] Feldmann H, Geisbert TW (2011). Ebola haemorrhagic fever. Lancet..

[26] Geisbert TW, Young HA, Jahrling PB, Davis KJ, Kagan E, Hensley LE (2003). Mechanisms underlying coagulation abnormalities in ebola hemorrhagic fever: overexpression of tissue factor in primate monocytes/macrophages is a key event. J Infect Dis..

[27] Okubo S, Miyamoto M, Takami K, Kanki M, Ono A, Nakatsu N (2013). Identification of novel liver-specific mRNAs in plasma for biomarkers of drug-induced liver injury and quantitative evaluation in rats treated with various hepatotoxic compounds. Toxicol Sci..

[28] Chertow DS, Kleine C, Edwards JK, Scaini R, Giuliani R, Sprecher A (2014). Ebola virus disease in West Africa--clinical manifestations and management. N Engl J Med..

[29] Schieffelin JS, Shaffer JG, Goba A, Gbakie M, Gire SK, Colubri A (2014). Clinical illness and outcomes in patients with Ebola in Sierra Leone. N Engl J Med..

[30] Lyon GM, Mehta AK, Varkey JB, Brantly K, Plyler L, McElroy AK (2014). Clinical care of two patients with Ebola virus disease in the United States. N Engl J Med..

[31] Rubins KH, Hensley LE, Wahl-Jensen V, Daddario DiCaprio KM, Young HA, Reed DS (2007). The temporal program of peripheral blood gene expression in the response of nonhuman primates to Ebola hemorrhagic fever. Genome Biol..

[32] Williams KJ, Qiu X, Fernando L, Jones SM, Alimonti JB (2015). VSVDeltaG/EBOV GP-induced innate protection enhances natural killer cell activity to increase survival in a lethal mouse adapted Ebola virus infection. Viral Immunol..

[33] Colubri A, Silver T, Fradet T, Retzepi K, Fry B, Sabeti P (2016). Transforming clinical data into actionable prognosis models: machine-learning framework and field-deployable app to predict outcome of Ebola patients. PLoS Negl Trop Dis..

[34] Cox E, Borio L, Temple R (2014). Evaluating Ebola therapies--the case for RCTs. N Engl J Med..

[35] Adebamowo C, Bah-Sow O, Binka F, Bruzzone R, Caplan A, Delfraissy JF (2014). Randomised controlled trials for Ebola: practical and ethical issues. Lancet..

[36] Joffe S (2014). Evaluating novel therapies during the Ebola epidemic. JAMA..

[37] Buggert M, Tauriainen J, Yamamoto T, Frederiksen J, Ivarsson MA, Michaelsson J (2014). T-bet and Eomes are differentially linked to the exhausted phenotype of CD8+ T cells in HIV infection. PLoS Pathog..

[38] Wahl-Jensen VM, Afanasieva TA, Seebach J, Ströher U, Feldmann H, Schnittler H-J (2005). Effects of Ebola virus glycoproteins on endothelial cell activation and barrier function. J Virol..

[39] Zaas AK, Chen M, Varkey J, Veldman T, Hero AO, Lucas J (2009). Gene expression signatures diagnose influenza and other symptomatic respiratory viral infections in humans. Cell Host Microbe..

[40] Garamszegi S, Yen JY, Honko AN, Geisbert JB, Rubins KH, Geisbert TW (2014). Transcriptional correlates of disease outcome in anticoagulant-treated non-human primates infected with ebolavirus. PLoS Negl Trop Dis..

[41] Tsalik EL, Henao R, Nichols M, Burke T, Ko ER, McClain MT (2016). Host gene expression classifiers diagnose acute respiratory illness etiology. Sci Transl Med.

[42] Martin M (2011). Cutadapt removes adapter sequences from high-throughput sequencing reads. EMBnet. journal..

[43] Kim D, Pertea G, Trapnell C, Pimentel H, Kelley R, Salzberg SL (2013). TopHat2: accurate alignment of transcriptomes in the presence of insertions, deletions and gene fusions. Genome Biol..

[44] Langmead B, Salzberg SL (2012). Fast gapped-read alignment with Bowtie 2. Nat Methods..

[45] Robinson MD, McCarthy DJ, Smyth GK (2010). edgeR: a Bioconductor package for differential expression analysis of digital gene expression data. Bioinformatics.

[46] Meyer D, Dimitriadou E, Hornik K, Weingessel A, Leisch F, Chang CC, et al. Support Vector Machines: The Interface to libsvm in package e1071. R package version 16-7. 2015. http://CRAN.R-project.org/package=e1071.

[47] Sing T, Sander O, Beerenwinkel N, Lengauer T (2005). ROCR: visualizing classifier performance in R. Bioinformatics..

[48] Villadangos JA, Shortman K (2010). Found in translation: the human equivalent of mouse CD8+ dendritic cells. J Exp Med..

[49] Haniffa M, Shin A, Bigley V, McGovern N, Teo P, See P (2012). Human tissues contain CD141hi cross-presenting dendritic cells with functional homology to mouse CD103+ nonlymphoid dendritic cells. Immunity..

[50] Auffray C, Sieweke MH, Geissmann F (2009). Blood monocytes: development, heterogeneity, and relationship with dendritic cells. Annu Rev Immunol..

