# Supplementary material for: Transcriptomic signatures differentiate survival from fatal outcomes in humans infected with Ebola virus
Source: Genome Biol. 2017 Jan 19;18:4. doi: 10.1186/s13059-016-1137-3 (PMC5244546; doi:10.1186/s13059-016-1137-3)
Supplement: Additional file 2: — Fold change of the abundance of selected cytokines showing comparison between fatal or survivor groups between humans (using our datasets) and non-human primate models of infection (using historical published data). (DOCX 184 kb) [file 13059_2016_1137_MOESM2_ESM.docx]

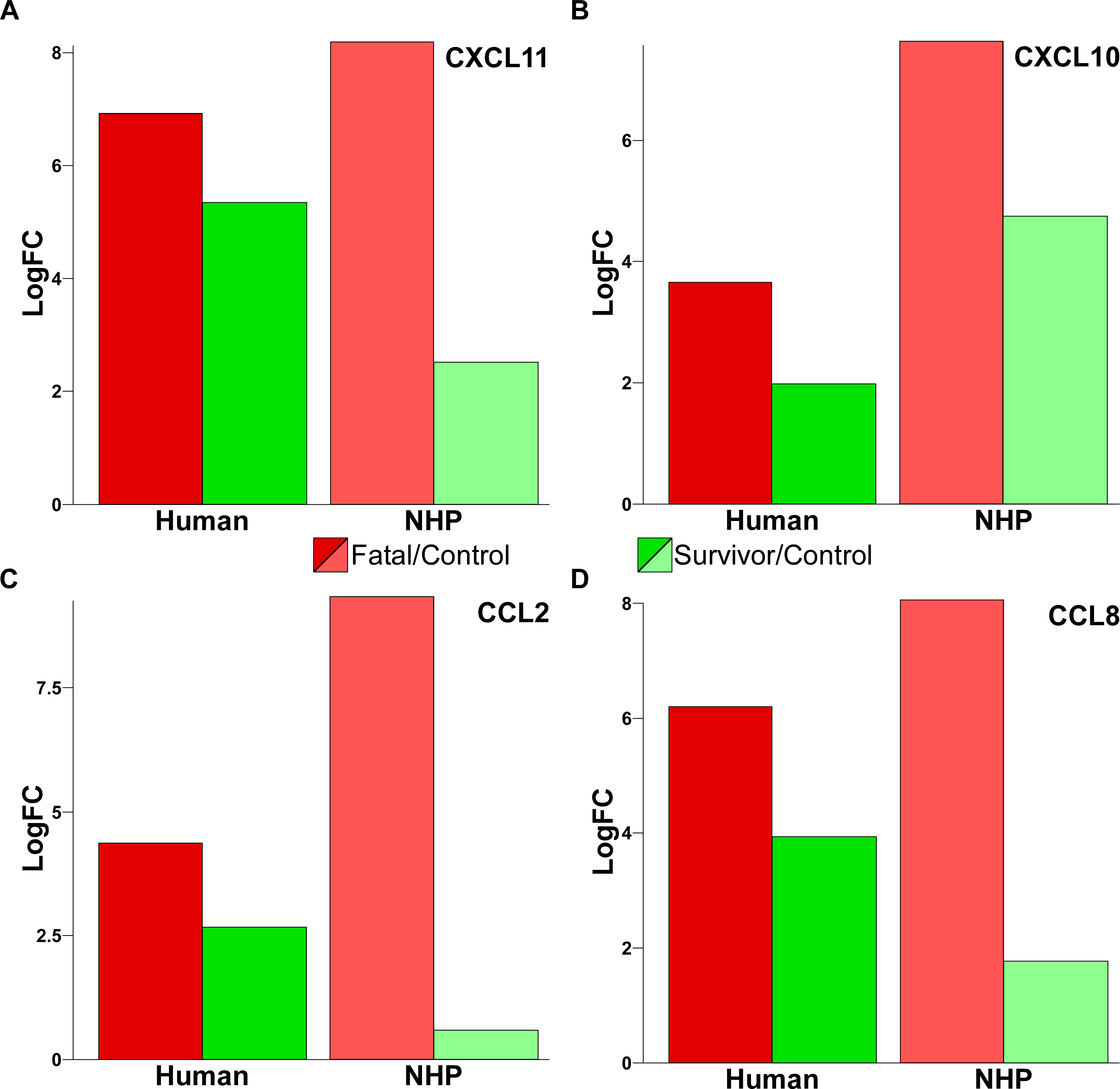


Differential accumulation of Cytokines during EBOV infection. A) bar plot illustrates the differential accumulation of CXCL11 mRNA in acute-fatal (dark red bar) and acute-survivor (green bar) human samples. For comparison, the differential accumulation of CXCL11 in non-human primates that succumb to EBOV infection is shown in the light red bar and the accumulation in individuals that survive EBOV infection is shown in the light green bar. Analogous data for CXCL10 is shown in B) for CCL2 in C) and for CCL8 in D). These plots indicate that mRNA changes seen in human infection are mimicked in primate models of lethal/non-lethal infection with EBOV.
