# Supplementary material for: Transcriptomic signatures differentiate survival from fatal outcomes in humans infected with Ebola virus
Source: Genome Biol. 2017 Jan 19;18:4. doi: 10.1186/s13059-016-1137-3 (PMC5244546; doi:10.1186/s13059-016-1137-3)
Supplement: Additional file 4: — Acute phase response genes activated in a non-human primate model of EBOV infection. (DOCX 159 kb) [file 13059_2016_1137_MOESM4_ESM.docx]

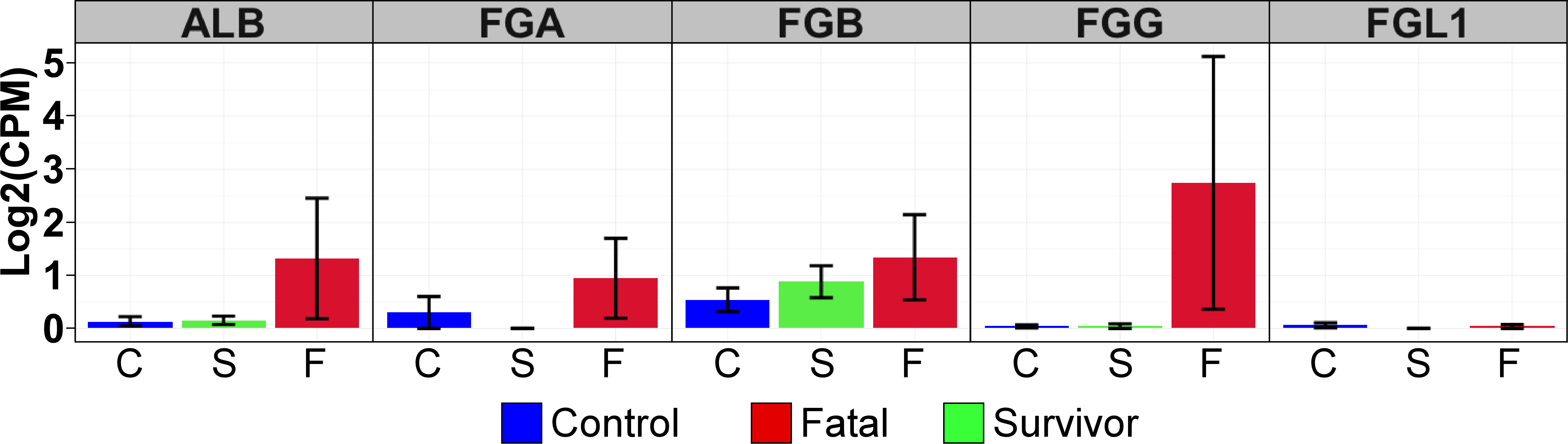


Acute phase response genes in NHPs infected with EBOV. All transcripts except FGL1 were detectable in the NHP model as well with an increase in transcripts associated with fatal disease vs survivors. For all groups, n=3.
