# Supplementary material for: Transcriptomic signatures differentiate survival from fatal outcomes in humans infected with Ebola virus
Source: Genome Biol. 2017 Jan 19;18:4. doi: 10.1186/s13059-016-1137-3 (PMC5244546; doi:10.1186/s13059-016-1137-3)
Supplement: Additional file 5: — DCQ analysis of predicted immune cell type profiles in acute patients who either went on to have a fatal outcome or survive infection. (DOCX 314 kb) [file 13059_2016_1137_MOESM5_ESM.docx]

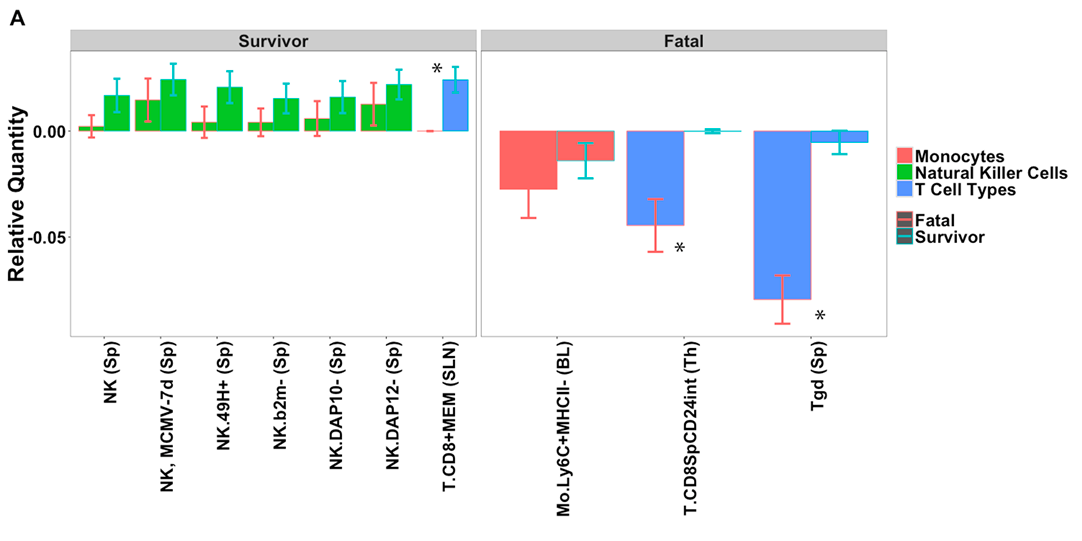


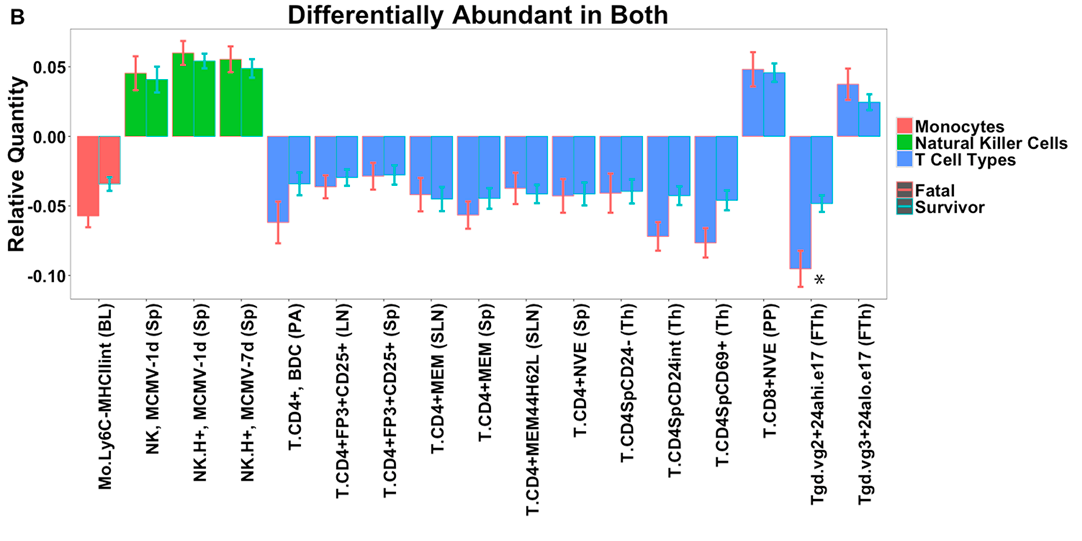


DCQ Results showing the mean relative abundance and sd. of significant cell type changes. A) represents cell types uniquely differentially abundant in acute-survivors (left) or acute-fatal (right) (statically greater than or less than 0 with p<0.05). The bars in red show specific monocytes, green is specific natural killer cell types and blue is specific T cell types. The bars with red error bars represent the mean and sd for acute-fatal and the bars with light blue error bars show the mean and sd for the acute survivors. Star indicates a significant difference between acute-fatal and acute-survivor with a p-value < 0.05. The specific cell type name is listed on the x-axis and the y-axis is the calculated relative abundance. B) is a similar plot for relative abundance of specific immune cell types but all cell types are significantly greater than or less than 0 in both acute-fatal and acute-survivor. Overall, in both acute-fatal and acute-survivor, there is a decrease in T cell types and dendritic cell types but an increase in natural killer cells.
