# Supplementary figures and images for: Transcriptomic signatures differentiate survival from fatal outcomes in humans infected with Ebola virus
Source: Genome Biol. 2017 Jan 19;18:4. doi: 10.1186/s13059-016-1137-3 (PMC5244546; doi:10.1186/s13059-016-1137-3)

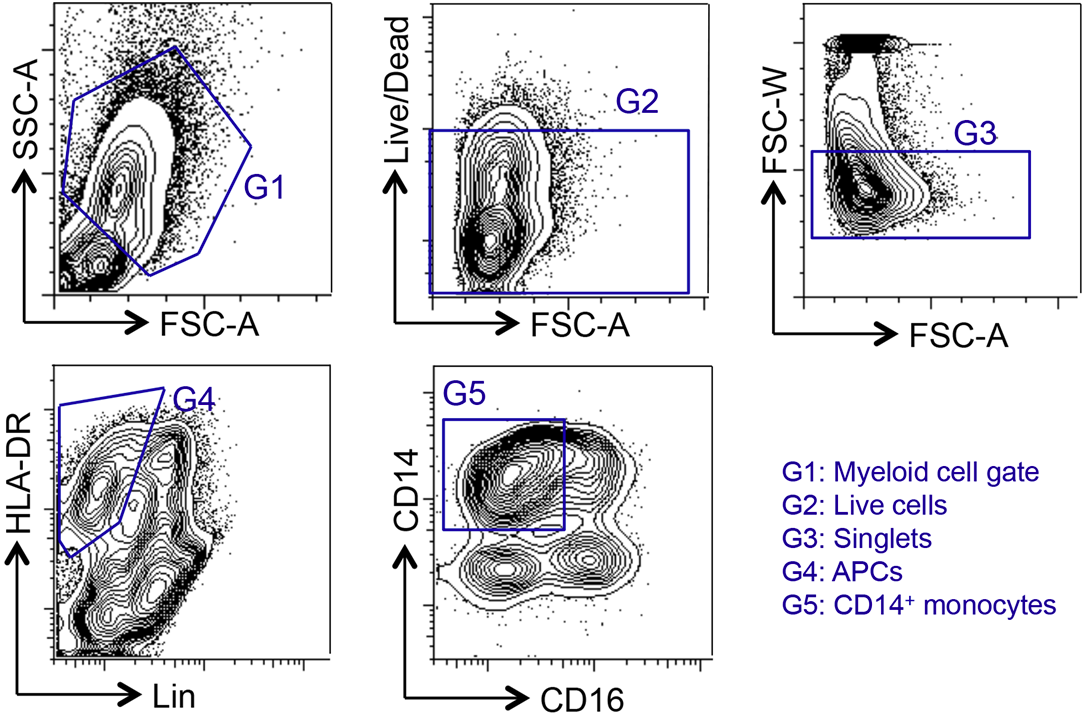


Flow cytometry gating strategy for discrimination of classic CD14+ peripheral blood monocytes.

Supplement: Additional file 6: — Flow cytometry gating strategy for discrimination of classic CD14+ peripheral blood monocytes. (DOCX 412 kb) [file 13059_2016_1137_MOESM6_ESM.docx]
