# Supplementary material for: Transcriptomic signatures differentiate survival from fatal outcomes in humans infected with Ebola virus
Source: Genome Biol. 2017 Jan 19;18:4. doi: 10.1186/s13059-016-1137-3 (PMC5244546; doi:10.1186/s13059-016-1137-3)
Supplement: Additional file 7: — Receiver operating characteristic (ROC) curves for p- value host classifier genes versus Ct value. (DOCX 49 kb) [file 13059_2016_1137_MOESM7_ESM.docx]

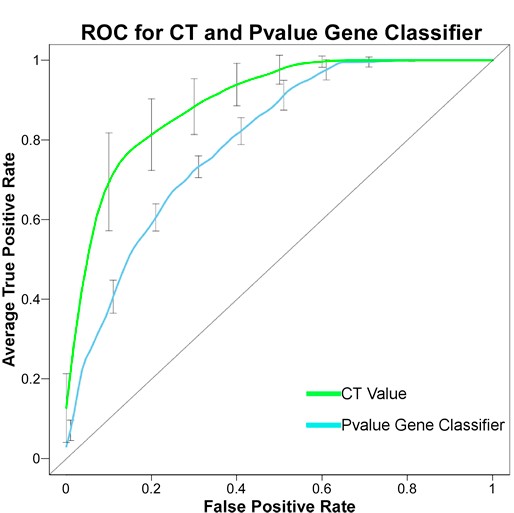


Receiver operating characteristic (ROC) curves for p- value host classifier genes. ROC curve for the CT value classifier (green) compared to the top 10 differentially expressed genes based on p-value (blue). On the x-axis is the false positive rate and the y-axis is the average true positive rate. The error bars represents the s.d. of the true positive rate. The line y=x represents where the classifier would fall it if was no better than randomly guessing. Overall, the p-value classifier did not perform better than the CT value.
