# Supplementary material for: Transcriptomic signatures differentiate survival from fatal outcomes in humans infected with Ebola virus
Source: Genome Biol. 2017 Jan 19;18:4. doi: 10.1186/s13059-016-1137-3 (PMC5244546; doi:10.1186/s13059-016-1137-3)
Supplement: Additional file 8: — Genes in the final profile sets for the machine learning approaches. (DOCX 103 kb) [file 13059_2016_1137_MOESM8_ESM.docx]

**Genes in the final profile genes set for the host classifier based on SVM.**

| Ensemble | Gene | Description |
| --- | --- | --- |
| ENSG00000163508 | EOMES | ISG, crucial for embryonic development of mesoderm and the central nervous system. May also be important in differentiation of effector CD8+ T cells |
| ENSG00000120708 | TGFBI | Inhibits cell adhesion |
| ENSG00000113758 | DBN1 | Cytoplasmic actin-binding protein that plays a role in neuronal growth |
| ENSG00000197153 | HIST1H3J | Core Component of nucleosome |
| ENSG00000143774 | GUK1 | Essential for recycling GMP and indirectly, cGMP |
| ENSG00000117114 | ADGRL2/LPHN2 | Regulates exocytosis |
| ENSG00000162692 | VCAM1 | ISG, member of the Ig superfamily and encodes a cell surface sialoglycoprotein expressed by cytokine-activated endothelium |
| ENSG00000171476 | HOPX | homeodomain protein that doesn't bind DNA, |
| ENSG00000162407 | PLPP/PPAP2B | Part of the PAP Family, membrane glycoprotein localized at the plasma membrane, important in receptor-activated signal transduction mediated by phosphlipase D |
| ENSG00000117228 | GBP1 | Induced by interferon, hydrolyzes GTP to GMP |

# Outcome of the “randomForest” classification method applied to the gene expression data generated in the Ebola project

OOB (Out Of Bag) error rate: 10.71% (accuracy = 89.29%)

Confusion matrix:

Fatal Survivor Class.error

(%)

Fatal 85 3 3.41

Survivor 9 15 37.50

# Identity of the genes used in the random forest-based classification routine

| Gene_id | Gene_name | Description |
| --- | --- | --- |
| ENSG0000022597 |  | mitochondrially encoded NADH:ubiquinone |
| 2 | MTND1P23 | oxidoreductase |
|  |  | core subunit 1 pseudogene 23 |
| ENSG0000025300 |  |  |
| 5 | RNU6-176P | RNA, U6 small nuclear 176, pseudogene |
| ENSG0000025500 |  |  |
| 2 |  | Non-annotated gene |
| ENSG0000025033 |  |  |
| 4 | LINC00989 | long intergenic non-protein coding RNA 989 |
| ENSG0000027097 | AC015849.1 |  |
| 7 | 6 | novel transcript |
| ENSG0000027150 |  |  |
| 3 | CCL5 | chemokine (C-C motif) ligand 5 |
| ENSG0000025701 |  |  |
| 7 | HP | haptoglobin |
| ENSG0000022975 |  | chemokine (C-X-C motif) receptor 2 |
| 4 | CXCR2P1 | pseudogene 1 |
| ENSG0000024139 |  |  |
| 9 | CD302 | CD302 molecule |
| ENSG0000010965 |  |  |
| 4 | TRIM2 | tripartite motif containing 2 |

# Summary outcome of the proposed profile-based classification method applied to the gene expression data generated in the Ebola project.

Accuracy estimated using leave-one-out validation approach: 91.96%

AUC (Area Under ROC Curve): 95.74%

Confusion matrix:

Fatal Survivor Class error

(%)

Fatal 84 4 4.55

Survivor 5 19 20.83

# Identity of the genes used in the profile-based classification routine

| Gene_id | Gene_symbol | Description |
| --- | --- | --- |
| ENSG00000162692 | VCAM1 | vascular cell adhesion molecule 1 |
| ENSG00000171476 | HOPX | HOP homeobox |
| ENSG00000131462 | TUBG1 | tubulin gamma 1 |
| ENSG00000162407 | PLPP3 | phospholipid phosphatase 3 |
| ENSG00000110079 | MS4A4A | membrane-spanning 4-domains, subfamily A, member 4A |
| ENSG00000120708 | TGFBI | transforming growth factor beta induced |
| ENSG00000100154 | TTC28 | tetratricopeptide repeat domain 28 |
| ENSG00000196290 | NF3L1 | NGG1 interacting factor 3 like 1 |
|  |  | solute carrier family 25 (mitochondrial carrier; adenine |
| ENSG00000005022 | SLC25A5 | nucleotide translocator), member 5 |
| ENSG00000135047 | CTSL | cathepsin L |
