# Supplementary material for: Transcriptomic signatures differentiate survival from fatal outcomes in humans infected with Ebola virus
Source: Genome Biol. 2017 Jan 19;18:4. doi: 10.1186/s13059-016-1137-3 (PMC5244546; doi:10.1186/s13059-016-1137-3)
Supplement: Additional file 9: — Outcome of predictive models for the Random Forest method. (PDF 201 kb) [file 13059_2016_1137_MOESM9_ESM.pdf]

Additional File 9

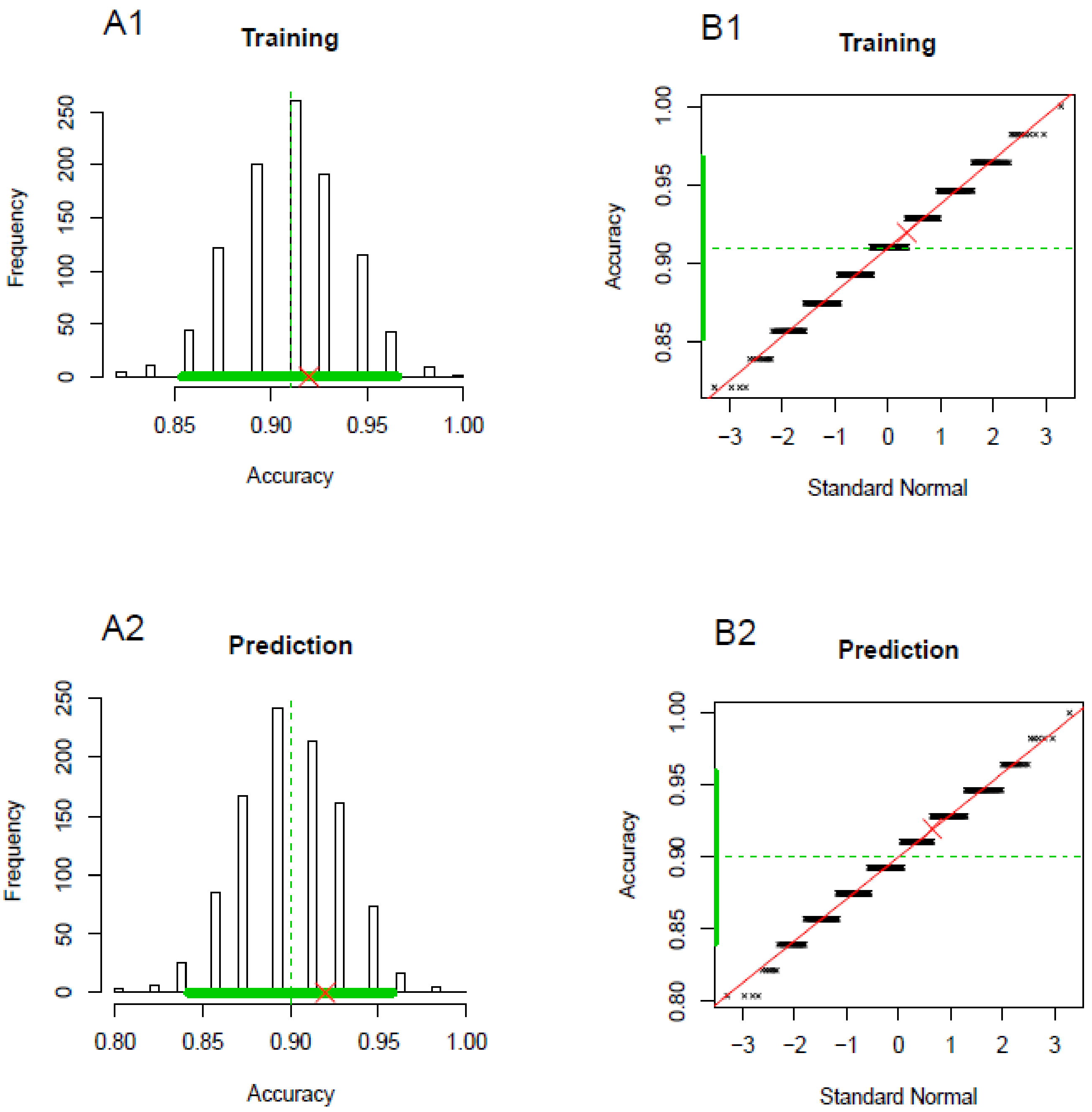

Figure Legend :

Outcome of predictive models for the Random Forest (RF) predictive method. Panels A1 and B1 indicate the frequency and accuracy distribution, respectively, of the resampling 1000 times of a training set comprising 50% of the entire dataset. Panels A2 and B2 displays the corresponding outcome of applying the training model to the remaining 50% of patients. For the A panels the dashed vertical line represents the mean value, and the thick horizontal green line represents the 95% confidence intervals (C.I.'s). The B panels present QQplots of accuracy as a function of a standard normal distribution for which a linear relationship is indicative of the data having a normal distribution. Again the thick and dashed thin green line have the same meaning as in panels A1 and 2.
