# Supplementary material for: Transcriptomic signatures differentiate survival from fatal outcomes in humans infected with Ebola virus
Source: Genome Biol. 2017 Jan 19;18:4. doi: 10.1186/s13059-016-1137-3 (PMC5244546; doi:10.1186/s13059-016-1137-3)
Supplement: Additional file 10: — The paired gene profiling method for outcome prediction. (PDF 386 kb) [file 13059_2016_1137_MOESM10_ESM.pdf]

Additional File 10

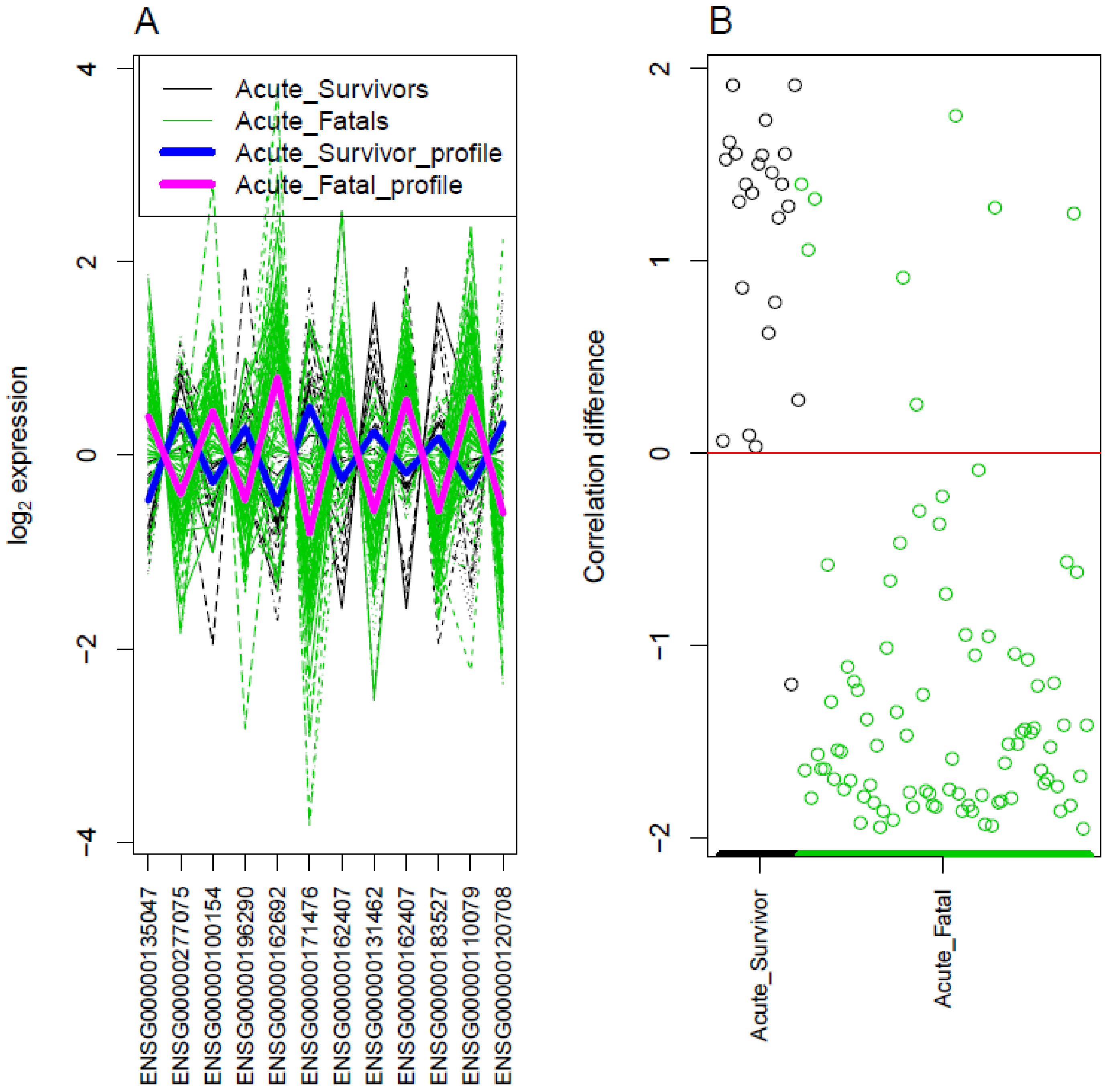

Figure Legend

The *paired gene profiling method* for outcome prediction. Gene pairs were selected by an algorithm searching through the gene space for those gene pairs displaying maximal discrimination between treatment groups. In this case 6 gene pairs were selected to provide a profile (left hand panel) from which the correlation coefficient (CC) of the training set for each of the two grouped profiles (heavy lines) was calculated. The CC difference for acute-fatal and acute-survivor patients were then displayed in order on the right hand panel. The red horizontal line separates the two domains, from which % prediction accuracy was calculated. Thus, the acute_survivor symbols (24) lie above the line with one exception, whilst for the 88 acute_fatal symbols 80 lay below the line and only 8 above.
