# Supplementary material for: Transcriptomic signatures differentiate survival from fatal outcomes in humans infected with Ebola virus
Source: Genome Biol. 2017 Jan 19;18:4. doi: 10.1186/s13059-016-1137-3 (PMC5244546; doi:10.1186/s13059-016-1137-3)
Supplement: Additional file 11: — A novel ‘gene paired profiling’ method for improved phenotypic prediction based on the optimised selection of representative multi-gene expression profiles. (DOCX 160 kb) [file 13059_2016_1137_MOESM11_ESM.docx]

**A novel ‘gene paired profiling’ method for improved phenotypic prediction based on the optimised selection of representative multi-gene expression profiles**

Gene expression profiles have been widely used for predicting phenotypic characteristics. The obvious precondition for achieving good performance of prediction is the availability of biomarker genes that can differentiate between the contrasted phenotypes. The most differentially expressed (DE, fold change and/or FDR) genes are frequently used as candidate biomarker genes. However, this does not guarantee satisfactory performance in a two- or multi-way classification, particularly if the expression distributions for each biomarker gene corresponding to phenotypes are substantially overlapping.

Suppose there are panels of genes across which their profiles of expression are sufficiently distinctive as to be associated in opposing ways with one or other of the two phenotypes. In this case the *Pearson product-moment correlation coefficient* offers a particularly direct predictor of which phenotype a particular profile is most closely associated. Thus, a query sample will be positively correlated with the profile established for one phenotype but negatively correlated with that established for the alternative phenotype. Indeed, maximizing the difference of the correlation coefficients for the two cases can be used as the metric for the initial selection of biomarker genes. Once identified, a biomarker profile can be built for each of the contrasting phenotypes. A query sample then can be judged based on the correlation of its expression profile with that established for each phenotypes.

We have formulated an optimization approach to the selection of biomarker gene which maximizes the distinctiveness of expression profiles across a panel of genes corresponding to each of two contrasting phenotypes. This has four steps - (i) assigning a ‘counteract’ score to each gene pair reflecting their opposing profile between these two phenotypes in the training set, and repeating this for all combinations of all gene models. Gene pairs with low counteract scores below an arbitrary cut-off threshold were then discarded, (ii) each of the remaining gene pairs were ordered by decreasing score, (iii) and for each phenotype and each gene pair, centering their expressions to zero, respectively, and finally (iv) deciding how many of the top-performing gene pairs should be incorporated into the biomarker profile based on performance they can achieve together. These four steps are detailed below.

Let T_1_ and T_2_ denote the two contrasting phenotypes, s_1_ and s_2_ denote the samples sizes for T_1_ and T_2_, and [g_i_ ,g_j_] denote a gene pair containing genes g_i_ and g_j_. Denote by e_1i_ and e_2i_ the expression vectors for gene g_i_ corresponding phenotypes T_1_ and T_2_, and similarly denote by e_1j_ and e_2j_ the expression vectors for gene g_j_ and respect to T_1_ and T_2_. In the first step, the counteract score of the the gene pair [g_i_ ,g_j_] is evaluated as:

Based on this formula, it is obvious that the score values are positive, and that the greater the score the more suited is the gene pair for discriminating between the two phenotypes. A default cut off threshold = 0.8 was used to extract discriminating gene pairs that were then ordered based on their decreasing scores for selection of gene pairs (step 2).

In step 3, for each of the gene pairs, their gene expression values were centred to zero with respect to each phenotype. This has the benefit of removing the impact of difference in relative expression levels presented between different gene pairs. The expression marker profile can then be constructed more effectively using multi-gene pairs.

Step 4 is a reiterative process in which the gene pairs that constitute the biomarker gene set are systematically altered to seek the best performing combination. In k^th^ iteration of the loop, k-1 gene pairs are included in profile marker genes, and are used to evaluate predictive performance based on the correlation coefficient. The performance for each additional gene pair to the growing panel is evaluated by calculation of the area under the curve (AUC) in a ROC analysis and the best one is compared with the performance of previous iteration. If the increase in performance is greater than the given criterion, the k+1 iteration is conducted, otherwise, the loop is terminated. On completion of the loop process the best performing gene pairs and the two biomarker profiles are obtained.

Now, for a query sample, the prediction can be conducted if only a measurement of its expression are available for each of the profiled biomarker genes pairs. Firstly, two expression values for each gene pair are centred to zero respectively; then correlation between query profile and defined marker profiles can be obtained. Finally, a prediction can be made based on the difference in two correlation coefficients of the query sample relative to the marker profiles.

An obvious and important advantage of this expression profile-based prediction method is its robustness, particularly as the prediction outcome is unaffected by any systematic shift or scale factor when using different sample batches or repeat measures. For example, a defined marker profile *M* and true query expression profile *Q* have a correlation coefficient *r* = *cor*(*M,Q*), when the existence of a shift *s* and scale factor *k* make the query profile becomes *k*Q+s ,* the results of the prediction will be unaffected, because *r* = *cor*(*M,Q*) = *co*r(*M,k*Q+s*).

Finally, this approach may be suitable for classification of two phenotypes, but not for classification of more than two categories.
