# Supplementary material for: Transcriptomic signatures differentiate survival from fatal outcomes in humans infected with Ebola virus
Source: Genome Biol. 2017 Jan 19;18:4. doi: 10.1186/s13059-016-1137-3 (PMC5244546; doi:10.1186/s13059-016-1137-3)
Supplement: Additional file 12: — Comparison of Ct values between the training and validation datasets. (DOCX 189 kb) [file 13059_2016_1137_MOESM12_ESM.docx]

Comparison of CT values in the two datasets. A) CT distributions for the initial dataset of 112 patients during acute disease. In read is the box and whisker plot of the fatal samples CT value and in green is the survivors. B is a similar plot but for a validation set of 20 patients. B is showing that in the validation dataset, the CT values for the patients shows not significance difference.
