# Supplementary material for: Transcriptomic signatures differentiate survival from fatal outcomes in humans infected with Ebola virus
Source: Genome Biol. 2017 Jan 19;18:4. doi: 10.1186/s13059-016-1137-3 (PMC5244546; doi:10.1186/s13059-016-1137-3)
Supplement: Additional file 13: — Gene set enrichment analysis on validation dataset. (DOCX 333 kb) [file 13059_2016_1137_MOESM13_ESM.docx]

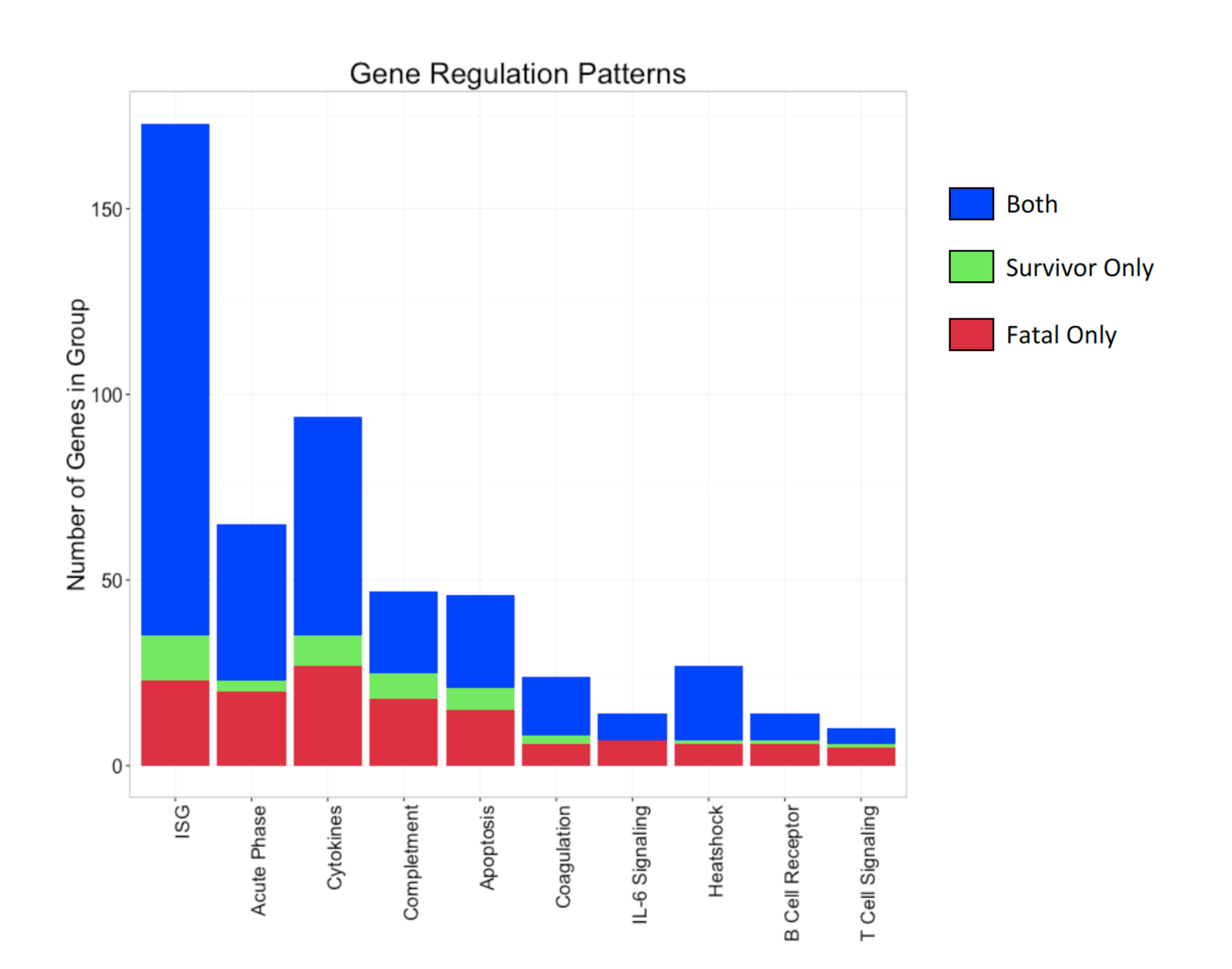


Gene set enrichment analysis on validation dataset. On the x axis is a given get set category with the y axis showing the number of genes in a given group that are significantly up-regulated compared to convalescence with genes in acute-survivor only in green, acute-fatal only in green and in both acute survivor and acute fatal in blue. In this gene set enrichment there is still a strong up-regulation of ISG, Acute Phase Genes, and Cytokines similar to the initial dataset.
