# Supplementary material for: Transcriptomic signatures differentiate survival from fatal outcomes in humans infected with Ebola virus
Source: Genome Biol. 2017 Jan 19;18:4. doi: 10.1186/s13059-016-1137-3 (PMC5244546; doi:10.1186/s13059-016-1137-3)
Supplement: Additional file 14: — Box and whisker plot for the top differentially expressed genes in validation dataset. (DOCX 134 kb) [file 13059_2016_1137_MOESM14_ESM.docx]

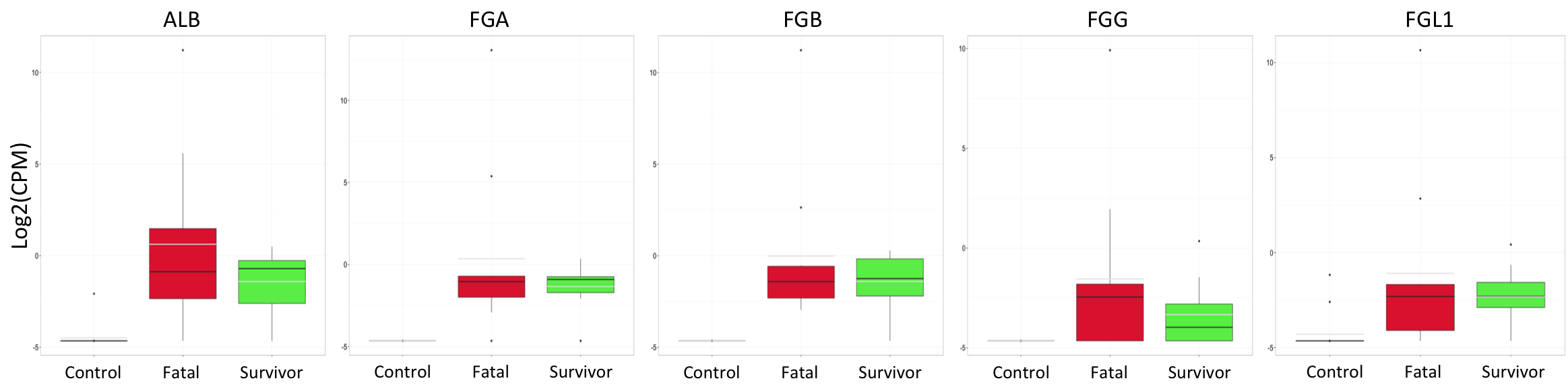


Box and whisker plot for the top differentially expressed genes in initial dataset in validation dataset with the acute fatal group shown in red, acute survivor in green, and convalescent survivors in blue. The black bar represents the median value. For all genes, there was significant increase in transcript abundance (log2(fold change) > 2 and FDR < 0.05) from convalescent survivors to acute disease (survivor or fatal). Also, in all genes except FFB there was a significant increase in transcript abundance in acute fatal compared to acute survivors.
